# Chondroprotective Effects of Enzyme-Treated Extract from *Cervus elaphus* L. in a Rat Model of Osteoarthritis

**DOI:** 10.3390/ijms27135785

**Published:** 2026-06-26

**Authors:** Min Ju Kim, Hyeon-Ji Lim, In-Sun Park, Bongsuk Choi, Taehee Kim, HyoungKwon Cho, Seon-Young Kim, Chan-Hun Jung

**Affiliations:** 1 Jeonju AgroBio-Materials Institute, Jeonju-si 54810, Jeollabuk-do, Republic of Korea; mjkim92@jami.re.kr (M.J.K.); lhj0923@jami.re.kr (H.-J.L.); witwit58@jami.re.kr (I.-S.P.); seon02@jami.re.kr (S.-Y.K.); 2 Hanpoong Pharmaceutical Co., Ltd., 301 Wanjusandan 6-ro, Bongdong-eup, Wanju-gun 55316, Jeollabuk-do, Republic of Korea; bongsuk333@hanpoong.co.kr (B.C.); taehee0317@naver.com (T.K.); hpoong@hanpoong.co.kr (H.C.)

**Keywords:** osteoarthritis, NP-2007, *Cervi cornu*, chondroprotective effect, COMP

## Abstract

Osteoarthritis (OA) is a chronic, debilitating degenerative joint disease whose prevalence is rising markedly with the rapid aging of the global population. In this study, we investigated the chondroprotective efficacy of NP-2007, an enzymatically hydrolyzed low-molecular-weight collagen from *Cervi cornu*, using IL-1β-stimulated SW1353 human chondrocyte cells and a medial meniscal transection (MMT)-induced OA rat model. In SW1353 cells, NP-2007 considerably suppressed the expression of inflammatory mediators (iNOS, COX-2) and cytokines (TNF-α, IL-6) without cytotoxicity. Crucially, it restored matrix homeostasis by downregulating catabolic enzymes (MMP-3, MMP-13, and ADAMTS-5) and upregulating anabolic markers (COL2A1, aggrecan), a process associated with the modulation of the Wnt/β-catenin and phosphoinositide 3-kinase/protein kinase B/mammalian target of rapamycin (PI3K/Akt/mTOR) signaling pathways and the recovery of the master chondrogenic factor SOX9. These in vitro findings were consistent with the in vivo results from the MMT model, where oral administration of NP-2007 (50 and 200 mg/kg) for 8 weeks effectively preserved articular cartilage structure and proteoglycan content while markedly reducing serum levels of catabolic biomarkers, including MMP-13 and COMP. Collectively, our findings demonstrate that NP-2007 exerts potent chondroprotective effects by modulating the balance between cartilage degradation and synthesis, suggesting its potential as a therapeutic candidate for the management of OA.

## 1. Introduction

Osteoarthritis (OA) is a chronic, debilitating degenerative joint disease characterized not merely by wear and tear but by a complex disruption of extracellular matrix (ECM) homeostasis [1]. The pathology is driven by the progressive attrition of articular cartilage, subchondral bone remodeling, and synovial inflammation, ultimately leading to joint failure [2]. With the rapidly aging global demographic, OA prevalence is on a steep upward trajectory, projected to affect nearly one billion individuals by 2050 [3]. Despite this growing burden, current pharmacological interventions remain largely palliative. While non-steroidal anti-inflammatory drugs and analgesics provide symptomatic relief, they fail to arrest the underlying structural progression of the disease and are frequently associated with gastrointestinal and cardiovascular toxicities upon chronic use [4]. Consequently, there is an urgent need to identify novel therapeutic agents capable of mitigating OA progression without significant adverse effects. In this context, recent research has extensively explored natural products, particularly traditional medicinal resources, as potential candidates for alternative and complementary OA therapies.

At the molecular level, OA pathogenesis is characterized by a profound metabolic transition within chondrocytes, where catabolic activities override anabolic processes [5]. This shift is frequently initiated by an inflammatory microenvironment dominated by cytokines such as interleukin-1β (IL-1β) [6]. Such stimuli trigger a network of intracellular signaling axes, notably the phosphoinositide 3-kinase/protein kinase B/mammalian target of rapamycin (PI3K/Akt/mTOR) pathways, which collectively drive the overexpression of matrix-degrading proteinases [7,8]. Crucial among these are matrix metalloproteinase (MMP)-3 and -13, responsible for the proteolysis of Type II collagen (COL2A1), and a disintegrin and metalloproteinase with thrombospondin motifs-5 (ADAMTS-5), a key aggrecanase that compromises cartilage elasticity by cleaving aggrecan [9]. This degradative cascade is further exacerbated by the downregulation of SOX9, a master transcription factor essential for the expression of matrix genes like *Col2a1* and *Acan* [10]. Therefore, therapeutic agents capable of simultaneously inhibiting these catabolic enzymes and restoring anabolic signaling represent a promising strategy for OA management.

Traditionally, deer antler (*Cervus elaphus* L.) has been utilized in medicinal practices for its potent bone-strengthening and tissue-regenerative properties [11,12]. However, the high molecular weight of its constituent proteins often restricts their intestinal absorption and systemic bioavailability. To circumvent these limitations, our previous research led to the development of a specialized enzyme-treated hydrolysate, designated as NP-2007, through a multi-step process employing both endogenous and exogenous proteases [13]. This enzymatic strategy yielded low-molecular-weight peptides characterized by specific bioactive sequences, such as GPAGERGSOGPAGPK and GPOGPAGPR, which are anticipated to exhibit superior tissue permeability and enhanced biological activity. Furthermore, we previously confirmed that NP-2007 exerts significant anti-inflammatory and analgesic effects in a monosodium iodoacetate (MIA)-induced rat model by effectively suppressing the expression of pro-inflammatory cytokines, including tumor necrosis factor (TNF)-α and IL-6 [13].

Although the MIA-induced model offers procedural simplicity and high reproducibility, it is limited by its relatively low correlation with the chronic, progressive degenerative alterations observed in human OA [14,15,16]. To overcome these constraints and rigorously validate the structural chondroprotective efficacy of NP-2007, the present study utilized the surgically induced medial meniscal transection (MMT) rat model. This model serves as a robust surrogate for the pathogenesis of human post-traumatic osteoarthritis (PTOA) and is specifically optimized for evaluating cartilage preservation and structural modifications within the joint architecture [17,18].

Therefore, in this study, we investigated the underlying molecular mechanisms of NP-2007 using SW1353 human chondrocyte cells, which are widely recognized as a standard model for evaluating IL-1β-induced catabolic signaling and matrix metalloproteinase expression due to their high reproducibility, and further validated its structural chondroprotective efficacy in an MMT rat model.

## 2. Results

### 2.1. NP-2007 Reduces Pro-Inflammatory Cytokine Production in SW1353 Cells

**Figure 1:**
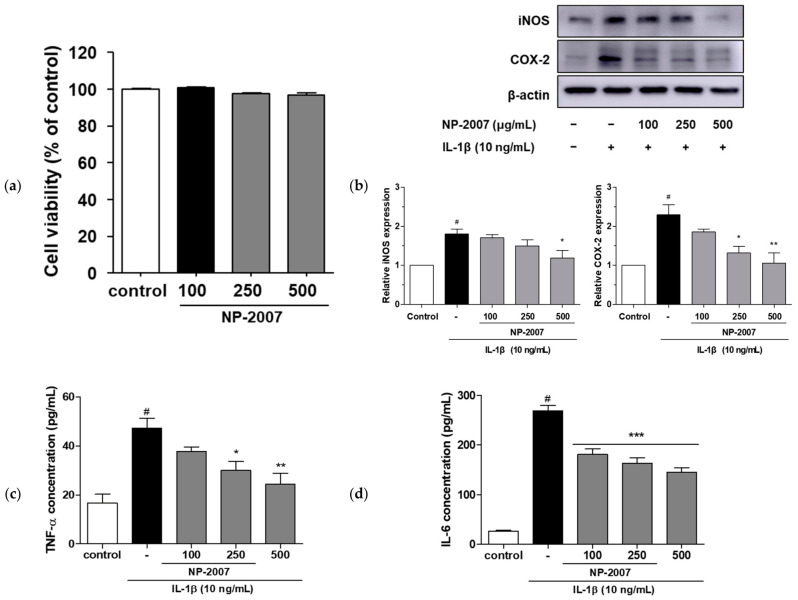
Effects of NP-2007 on cell viability and IL-1β-induced inflammatory responses in SW1353 cells. (**a**) Cytotoxic effect of NP-2007 in SW1353 cells measured by EZ-CYTOX assay. (**b**) Protein expression of iNOS and COX2 was measured by Western blotting (**top**) and quantified through densitometric analysis normalized to β-actin (**bottom**). (**c**) TNF-α and (**d**) IL-6 secretion were determined by ELISA. Compared with the control, # *p* < 0.05; compared with the IL-1β-treated group, * *p* < 0.05, ** *p* < 0.01, and *** *p* < 0.001. IL-1β, interleukin-1 beta; EZ-CYTOX, cell viability assay kit; iNOS, inducible nitric oxide synthase; COX2, cyclooxygenase-2; TNF-α, tumor necrosis factor alpha; IL-6, interleukin-6; ELISA, enzyme-linked immunosorbent assay.

To assess the potential cytotoxic effects of NP-2007 on human chondrocyte cells, SW1353 cells were treated with various concentrations (100, 250, and 500 µg/mL) for 24 h. As shown in Figure 1a, cell viability remained unaffected by NP-2007 at concentrations up to 500 µg/mL, indicating that the treatment was non-cytotoxic. Subsequently, we investigated whether NP-2007 could suppress the inflammatory response in SW1353 cells under these non-cytotoxic conditions. To mimic the catabolic environment of OA, cells were stimulated with IL-1β (10 ng/mL). This stimulation markedly induced the protein expression of inducible nitric oxide synthase (iNOS) and cyclooxygenase-2 (COX-2), which are critical inflammatory mediators. However, treatment with NP-2007 effectively attenuated the expression of both iNOS and COX-2 in a dose-dependent manner (Figure 1b). Furthermore, the effects of NP-2007 on the production of pro-inflammatory cytokines, specifically TNF-α and IL-6, were evaluated via enzyme-linked immunosorbent assay (ELISA). IL-1β treatment considerably upregulated the secretion of these cytokines compared to the untreated control group. In contrast, NP-2007 significantly suppressed the production of both TNF-α and IL-6, exhibiting a clear dose–response tendency (Figure 1c,d). Collectively, these findings demonstrate that NP-2007 exerts potent anti-inflammatory effects in SW1353 human chondrocyte cells by downregulating the expression of pro-inflammatory cytokines.

### 2.2. NP-2007 Modulates the Balance Between Catabolic and Anabolic Factors in IL-1β-Induced SW1353 Cells

**Figure 2:**
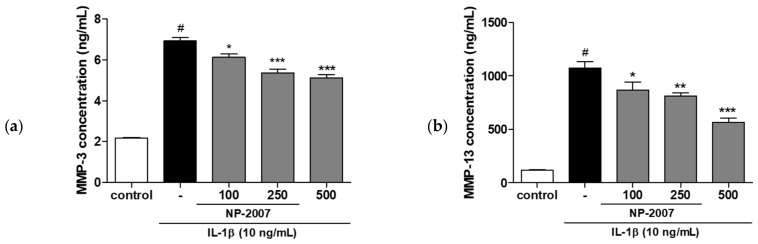

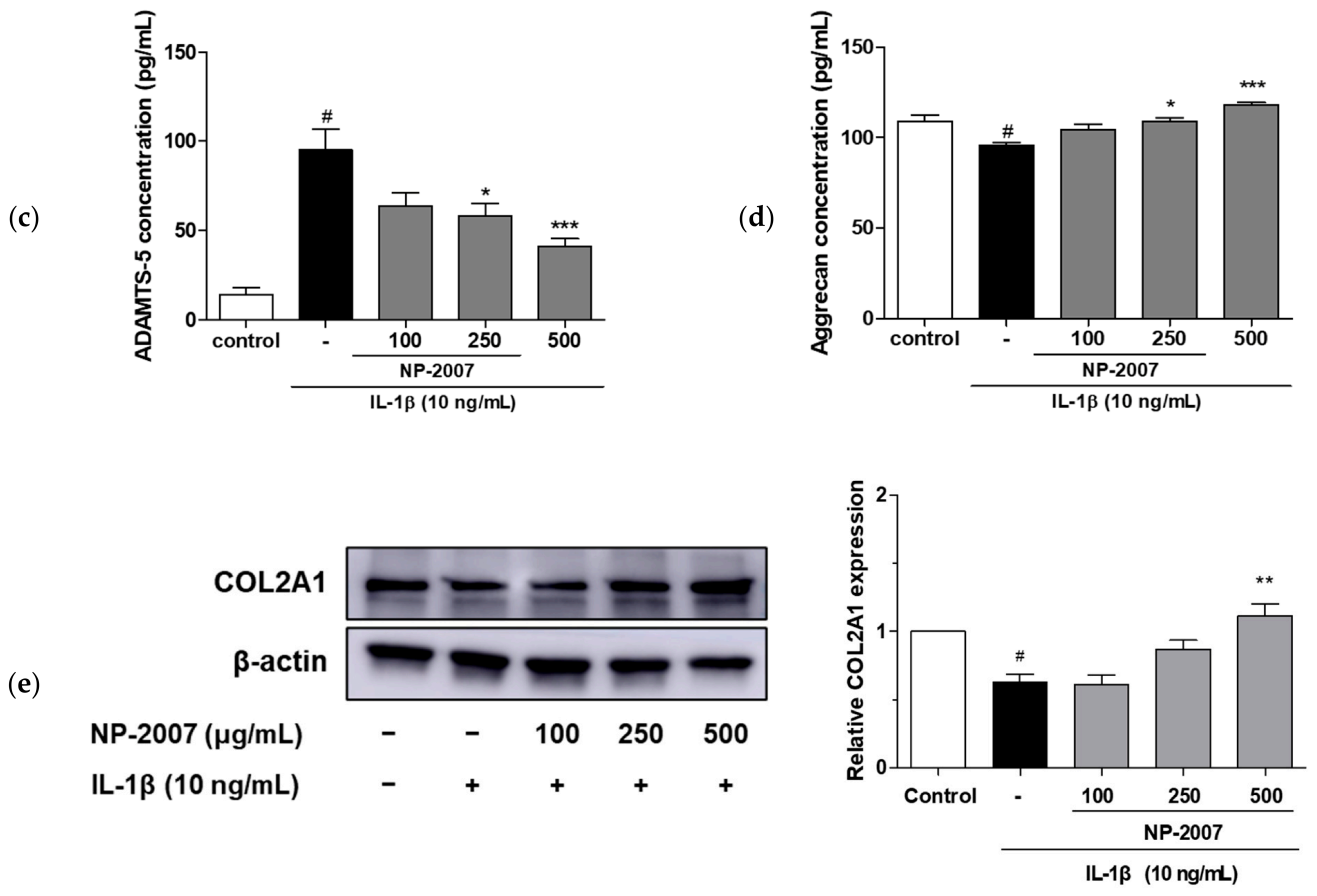
Effects of NP-2007 on matrix homeostasis in IL-1β-stimulated SW1353 cells. Secretion of (**a**) MMP-3, (**b**) MMP-13, (**c**) ADAMTS-5, and (**d**) aggrecan was determined by ELISA. (**e**) The protein expression levels of COL2A1 were measured by Western blotting (**left**) and quantified through densitometric analysis normalized to β-actin (**right**). Compared with the control, # *p* < 0.05; compared with the IL-1β-treated group, * *p* < 0.05, ** *p* < 0.01, and *** *p* < 0.001. MMP-3, matrix metalloproteinase-3; MMP-13, matrix metalloproteinase-13; ADAMTS-5, a disintegrin and metalloproteinase with thrombospondin motifs-5; COL2A1, collagen type II alpha 1 chain.

To investigate whether NP-2007 regulates the imbalance between catabolic and anabolic factors associated with OA progression, SW1353 cells were stimulated with IL-1β (10 ng/mL) and treated with NP-2007 at various concentrations (100, 250, and 500 μg/mL). First, we assessed the expression of matrix-degrading enzymes. As shown in Figure 2a,b, IL-1β stimulation markedly upregulated the secretion of MMP-3 and MMP-13, which are pivotal enzymes driving the cleavage of proteoglycans and COL2A1, respectively. However, treatment with NP-2007 considerably attenuated the IL-1β-induced upregulation of MMP-3 and MMP-13 in a dose-dependent manner. Consistent with these findings, NP-2007 also effectively suppressed the elevated levels of ADAMTS-5, the primary aggrecanase implicated in the early stages of cartilage erosion (Figure 2c). Subsequently, we evaluated the protective effect of NP-2007 on the structural integrity of the ECM by measuring the levels of aggrecan and COL2A1. IL-1β stimulation markedly reduced the protein levels of aggrecan and COL2A1, indicating a disruption in matrix homeostasis (Figure 2d,e). Notably, treatment with NP-2007 effectively restored the expression of these major structural proteins in a dose-dependent manner. At the highest concentration (500 μg/mL), aggrecan and COL2A1 expression levels were recovered to levels comparable to or even exceeding those of the untreated control group. Collectively, these results demonstrate that NP-2007 exerts a potent chondroprotective effect via a dual mechanism: inhibiting the expression of catabolic enzymes (MMP-3, MMP-13, and ADAMTS-5) and preserving the anabolic synthesis of major cartilage matrix proteins (aggrecan, COL2A1).

### 2.3. NP-2007 Modulates β-Catenin/GSK3β and PI3K/Akt/mTOR Signaling Pathways in IL-1β-Induced SW1353 Cells

**Figure 3:**
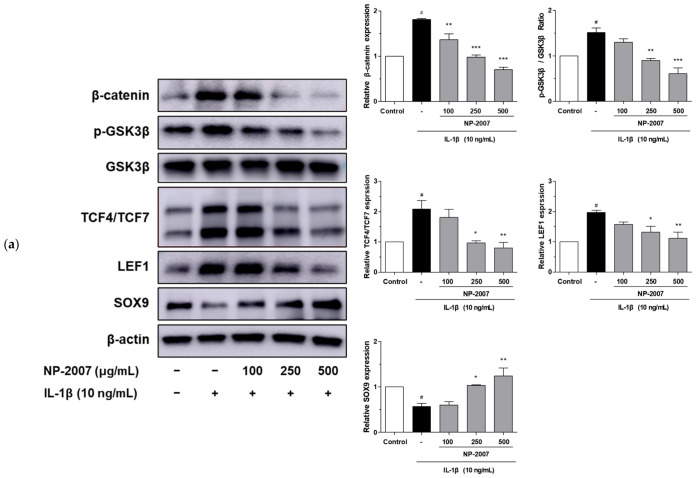

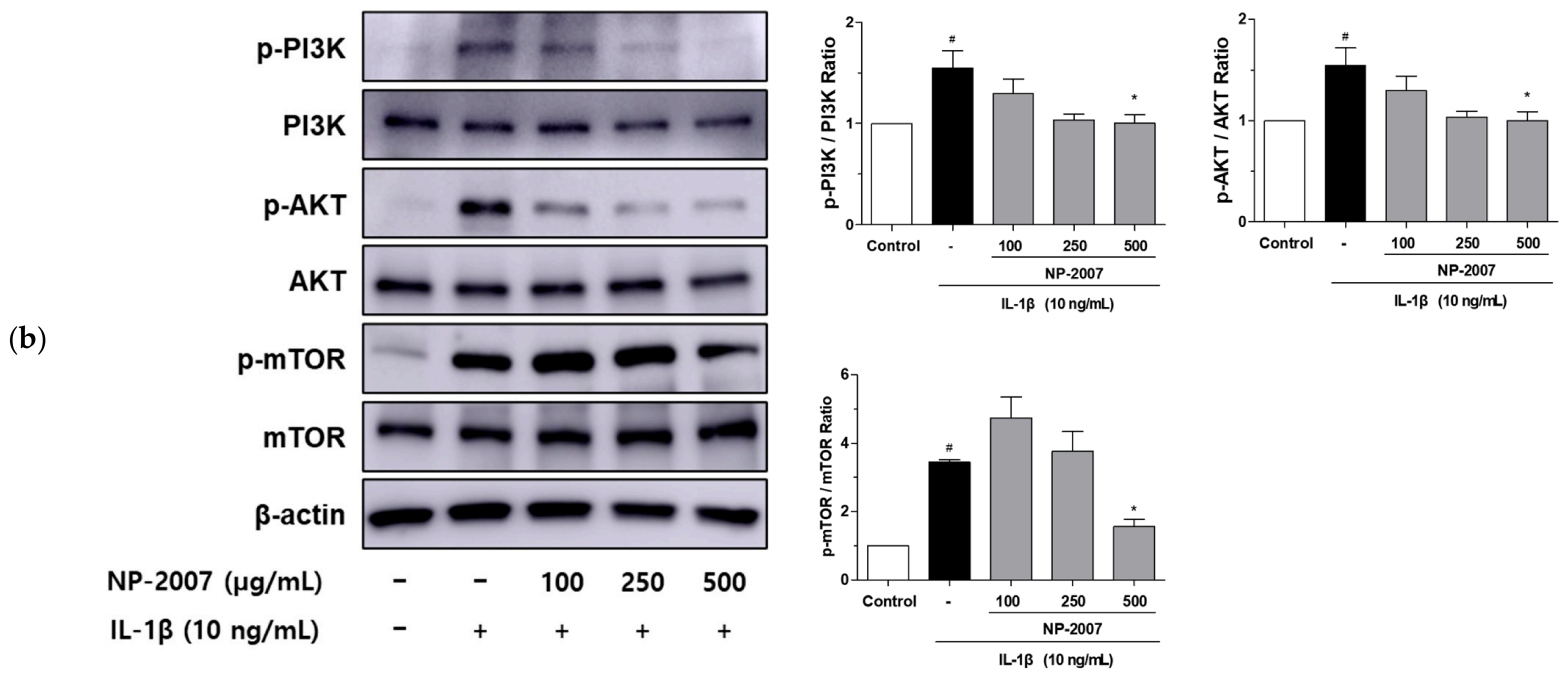
Effect of NP-2007 on β-catenin/GSK3β and PI3K/Akt/mTOR signaling pathways in IL-1β-stimulated SW1353 cells. (**a**) β-catenin/GSK3β signaling pathway markers and SOX9 were measured by Western blotting (**left**), and quantified through densitometric analysis normalized to β-actin, whereas p-GSK3β was normalized to total GSK3β (**right**). (**b**) PI3K/Akt/mTOR signaling pathways markers were measured by western blotting (**left**), and quantified through densitometric analysis normalized to their respective total proteins (**right**). Compared with the control, # *p* < 0.05; compared with the IL-1β-treated group, * *p* < 0.05, ** *p* < 0.01, and *** *p* < 0.001.

To elucidate the molecular mechanisms governing the chondroprotective and anti-inflammatory effects of NP-2007, we investigated its impact on the Wnt/β-catenin and PI3K/Akt/mTOR signaling pathways, both of which play pivotal roles in OA pathogenesis. As shown in Figure 3a, stimulation with IL-1β (10 ng/mL) markedly activated the canonical Wnt pathways, as evidenced by the accumulation of β-catenin and the upregulation of its downstream transcription factors, TCF4/TCF7 and LEF1. Furthermore, IL-1β treatment increased the phosphorylation of GSK-3β, which inactivates the kinase and prevents the proteasomal degradation of β-catenin. However, treatment with NP-2007 effectively reversed these pathological changes in a dose-dependent manner. NP-2007 downregulated the protein levels of β-catenin, TCF4/TCF7, and LEF1 while simultaneously suppressing GSK-3 β phosphorylation, suggesting that it promotes β-catenin degradation by restoring GSK-3β activity. In parallel, we examined the PI3K/Akt/mTOR signaling cascade (Figure 3b), a critical axis for regulating cellular metabolism and inflammatory responses. IL-1β stimulation markedly induced the phosphorylation of PI3K, Akt, and mTOR, indicating robust pathway activation. Treatment with NP-2007 considerably attenuated the phosphorylation of these kinases in a dose-dependent manner (100, 250, and 500 μg/mL). Specifically, at the 500 μg/mL concentration, the phosphorylation levels were reduced to near-basal levels, demonstrating the potent inhibitory effect of NP-2007 on this inflammatory cascade. Crucially, the modulation of these pathways was associated with the recovery of SOX9, the master transcription factor for chondrogenesis. While IL-1β notably suppressed SOX9 expression, NP-2007 treatment remarkably restored its levels (Figure 3a). This restoration of SOX9 is consistent with the previously observed upregulation of COL2A1 and aggrecan (Figure 2). Collectively, these findings suggest that the chondroprotective effects of NP-2007 may be mediated, at least in part, by the modulation of the catabolic Wnt/β-catenin and PI3K/Akt/mTOR pathways while enhancing the anabolic SOX9 signaling axis.

### 2.4. NP-2007 Prevents Cartilage Degradation and Improves Histological Changes in the MMT-Induced OA Rat Model

**Figure 4:**
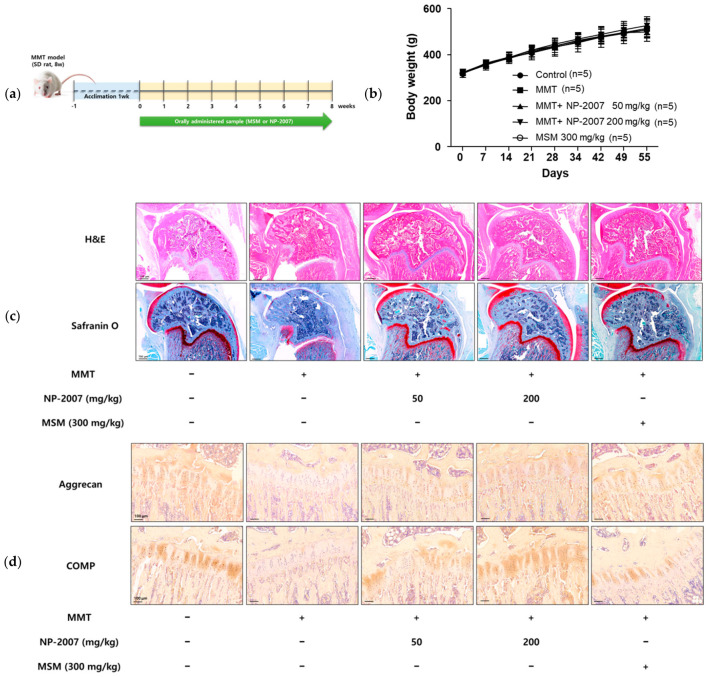
Effect of NP-2007 on histopathological and immunohistochemical changes from knee joint tissue of MMT-induced OA rats. (**a**) Experimental schedule. (**b**) Body weight changes during the experimental period. (**c**) Histopathological analysis of knee joint tissue by H&E and safranin O staining. (**d**) Immunohistochemical analysis of knee joint tissue. MMT, medial meniscal transection; OA, osteoarthritis; H&E, hematoxylin and eosin.

To evaluate the in vivo therapeutic efficacy of NP-2007, we employed the MMT-induced OA rat model, which mimics the structural progression of human PTOA. Following MMT surgery, rats were orally administered NP-2007 (50 and 200 mg/kg) or methylsulfonylmethane (MSM; 300 mg/kg) for 8 weeks (Figure 4a). Throughout the experimental period, no significant differences in body weight gain were observed among all groups, suggesting that NP-2007 does not induce overt systemic toxicity at the doses tested (Figure 4b). Histopathological analysis of the knee joints was performed using Hematoxylin and Eosin (H&E) and Safranin O staining to assess structural integrity and proteoglycan content. As shown in Figure 4c, the MMT group exhibited severe cartilage attrition, irregular articular surfaces, and a marked reduction in Safranin O staining intensity, indicating extensive proteoglycan depletion. In contrast, treatment with NP-2007 markedly preserved the articular surface and prevented the loss of proteoglycans. Notably, the 200 mg/kg NP-2007 group showed a remarkable restoration of the cartilage matrix, comparable to the MSM-treated positive control group. To further characterize the chondroprotective effect of NP-2007 at the molecular level, immunohistochemical (IHC) staining for aggrecan and cartilage oligomeric matrix protein (COMP) was conducted (Figure 4d). The MMT group showed a drastic decrease in the expression of both aggrecan and COMP within the articular cartilage, reflecting the catabolic destruction of the ECM. However, the administration of NP-2007 effectively inhibited the degradation of these essential structural proteins in a dose-dependent manner. Specifically, the 200 mg/kg dose considerably maintained the expression of aggrecan and COMP, reinforcing the structural stability of the cartilage ECM. Collectively, these histological and IHC findings demonstrate that NP-2007 possesses potent in vivo chondroprotective properties by mitigating cartilage erosion and maintaining matrix homeostasis in the mechanically induced OA model.

### 2.5. NP-2007 Suppresses Serum Levels of Matrix-Degrading Enzymes and Structural Biomarkers in MMT-Induced OA Rats

**Figure 5:**
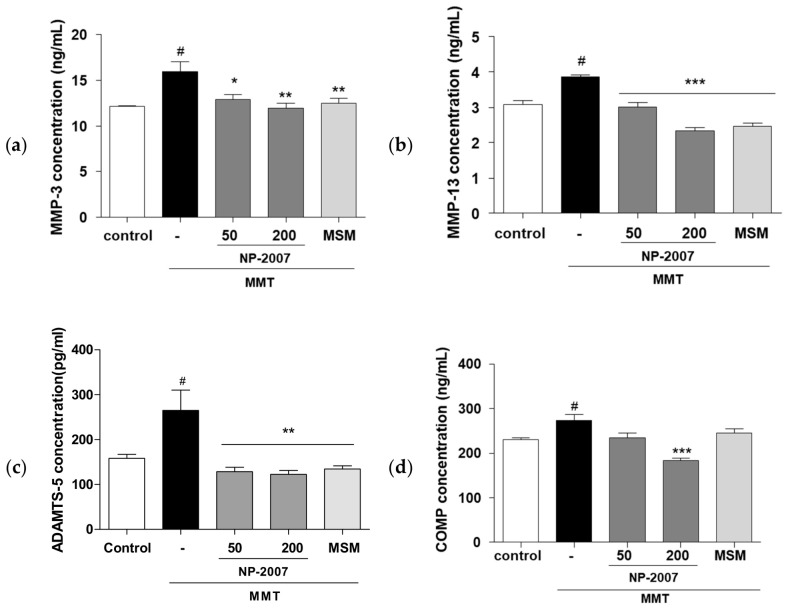
Effect of NP-2007 on matrix-degrading enzymes and structural biomarkers production in the serum of MMT-induced OA rats. (**a**) MMP-3, (**b**) MMP-13, (**c**) ADAMTS-5, and (**d**) COMP levels were measured by ELISA. Compared with the control, # *p* < 0.05; compared with the MMT-induced group, * *p* < 0.05, ** *p* < 0.01, and *** *p* < 0.001. COMP, cartilage oligomeric matrix protein.

To validate the biochemical impact of NP-2007 on cartilage metabolism, we analyzed the serum concentrations of key matrix-degrading enzymes and turnover markers. In the MMT-induced OA model, the activities of enzymes that drive the degradation of collagen and proteoglycans are typically upregulated. As shown in Figure 5a,b, the serum levels of MMP-3 and MMP-13 were markedly increased in the MMT group compared to those in the control group. Oral administration of NP-2007 (50 and 200 mg/kg) considerably attenuated the elevation of these enzymes. Notably, the 200 mg/kg dose showed a more substantial reduction in MMP-13 levels than the 50 mg/kg dose and was comparable to that of the MSM (300 mg/kg) positive control. Furthermore, we examined ADAMTS-5, the primary aggrecanase implicated in early cartilage erosion. The surgical induction of OA markedly elevated serum ADAMTS-5 levels, whereas NP-2007 treatment effectively suppressed this increase (Figure 5c). We also measured COMP, which is released into the circulation during cartilage matrix breakdown and serves as a sensitive indicator of joint damage. Serum COMP concentration considerably increased in the MMT group but was dose-dependently reduced by NP-2007 treatment, with the 200 mg/kg dose showing the most significant inhibitory effect (Figure 5d). These results are consistent with our previous findings that NP-2007, a low-molecular-weight collagen hydrolysate, suppresses matrix metalloproteinases to preserve joint architecture. Collectively, these biochemical data demonstrate that NP-2007 effectively inhibits the systemic expression of catabolic biomarkers, thereby protecting the structural integrity of the knee joint in a mechanically induced OA model.

## 3. Discussion

Given the escalating global burden of OA, there is a critical surge in research aimed at developing therapeutic strategies that transcend the limitations of current palliative care [3,19]. Recent studies are increasingly focusing on natural products with diverse bioactivities and superior safety profiles to investigate therapeutic strategies that maintain cartilage integrity and arrest catabolic progression without serious side effects [20,21,22]. Our research group developed NP-2007, an enzyme-treated hydrolyzed low-molecular-weight collagen derived from *Cervi cornu*, and has conducted a series of investigations to evaluate its therapeutic potential [13]. While our previous study established the potent anti-inflammatory and analgesic effects of NP-2007 in an MIA-induced OA model, the present study focused on confirming its direct chondroprotective efficacy [13]. In this study, we demonstrated that NP-2007 effectively restores metabolic balance in SW1353 human chondrocyte cells and exerts substantial chondroprotective effects in an MMT-induced OA rat model, thereby suggesting its potential as a promising therapeutic agent capable of inhibiting structural joint degeneration.

OA is a degenerative joint disease characterized by the progressive destruction of ECM due to an imbalance between anabolic and catabolic processes within chondrocytes [23,24]. The initiation and progression of OA are fundamentally driven by the inflammatory environment within the intra-articular space [25]. In particular, catabolism in chondrocytes is known to be triggered by pro-inflammatory cytokines, especially IL-1β, which acts as a primary mediator in the degradation of articular cartilage [26]. IL-1β induces the catabolic phase of OA by upregulating the expression of the inflammatory mediators iNOS and COX-2, as well as the pro-inflammatory cytokines TNF-α and IL-6, in chondrocytes [26,27]. This inflammatory cascade subsequently orchestrates the breakdown of the joint architecture by triggering the overproduction of various matrix-degrading enzymes. Among these, MMPs—specifically MMP-3 and MMP-13—play a decisive role in the irreversible cleavage of the COL2A1 framework through both direct proteolytic action and indirect activation of downstream catabolic cascades [9,28]. Concurrently, ADAMTS-5 acts as the major aggrecanase responsible for the early depletion of proteoglycans, which ultimately compromises cartilage elasticity and structural integrity [29]. Our previous study demonstrated that NP-2007 shows an inhibitory effect on inflammatory response by reducing the expression of the inflammatory mediators iNOS and COX-2, as well as the pro-inflammatory cytokines TNF-α and IL-6, and decreased joint cartilage loss by regulating MMPs in lipopolysaccharide-induced Raw 264.7 cells [13]. In this study, we induced the catabolic phase of OA in SW1353 human chondrocyte cells by treatment with IL-1β and investigated the inhibitory effects of NP-2007 on inflammatory response and its regulatory effects on MMP expression. We observed that IL-1β induced the expression of inflammatory mediators (iNOS and COX-2) and pro-inflammatory cytokines (TNF-α and IL-6) while increasing the secretion of MMP-3, MMP-13, and ADAMTS5, which consequently led to a decrease in the expression of aggrecan and COL2A1. We confirmed that NP-2007 effectively inhibited these IL-1β-induced inflammatory factors. Consequently, the expression of aggrecan and COL2A1, the primary structural components of the chondrocyte ECM, was restored by NP-2007 treatment.

Although the molecular pathogenesis of OA has not been fully elucidated, recent evidence suggests that the nuclear factor kappa B (NF-κB), mitogen-activated protein kinase (MAPK), Wnt/β-catenin, and PI3K/Akt/mTOR signaling pathways are closely associated with cartilage degeneration [30,31,32]. These pathways are known to be triggered by IL-1β, a key proinflammatory cytokine in the OA microenvironment [26]. Our previous study demonstrated that NP-2007 attenuates the progression of OA by regulating the NF-κB and MAPK signaling pathways [13]. Thus, the present study investigated whether NP-2007 also modulates other pivotal cascades, specifically the Wnt/β-catenin and PI3K/Akt/mTOR signaling pathways. In the canonical Wnt pathway, β-catenin translocates from the cytoplasm to the nucleus, where it induces the transcription of downstream target genes such as TCF/LEF [32,33]. This process promotes the expression of catabolic enzymes (MMPs and ADAMTSs) and facilitates cartilage degradation, partly by suppressing SOX9 expression [34]. GSK3β acts as a key regulator of this pathway by targeting β-catenin for degradation; however, IL-1β stimulation induces the inhibitory phosphorylation of GSK3β, thereby stabilizing β-catenin and allowing its nuclear accumulation [33,35]. Furthermore, the PI3K/Akt/mTOR signaling pathway is essential for maintaining cartilage homeostasis, yet its overactivation by IL-1β is known to accelerate cartilage degeneration [32]. In this study, we observed that IL-1β treatment sequentially induced the phosphorylation of GSK3β, the accumulation of β-catenin, and the upregulation of TCF4/TCF7 and LEF1. Consequently, the expression of SOX9, a master transcription factor for cartilage development and maintenance, was markedly reduced. We also confirmed that the PI3K/AKT/mTOR signaling axis was activated upon IL-1β treatment. Notably, NP-2007 effectively inhibited the IL-1β-induced phosphorylation of GSK3β and the subsequent activation of the β-catenin/TCF/LEF complex. As a result, the expression of SOX9 was markedly restored by NP-2007 treatment, suggesting that NP-2007 preserves the chondrocyte phenotype by rebalancing these critical signaling networks.

Animal models of OA are broadly classified into spontaneous models, which develop naturally with aging and genetic manipulation, and induced models, which are triggered by external stimuli [36]. Most laboratories utilize induced models because they can replicate OA pathology within a relatively short timeframe [37]. These are further stratified into surgically induced, chemically induced, and non-invasive mechanical stimulation models [36]. Surgically induced models, such as the MMT model, involve the resection or transection of the medial meniscus to induce joint instability. The MMT model effectively mimics the pathogenesis of human PTOA and is primarily used to evaluate chondroprotective efficacy and structural joint changes [17,18,38]. In contrast, chemically induced models, typically established by intra-articular MIA injection, offer ease of induction but are limited by their low correlation with the progressive degenerative changes observed in human OA [14,15]. Since MIA primarily induces rapid chondrocyte death and acute inflammation, it is more predictive of analgesic drug efficacy rather than structural modification [16].

Therefore, in this study, we determined the chondroprotective potential of NP-2007 using the MMT-induced OA rat model to specifically observe its impact on cartilage preservation and structural integrity. In our MMT-induced group, we observed severe cartilage damage accompanied by a marked loss of aggrecan and COMP, both of which are critical structural components of the ECM. However, the administration of NP-2007 considerably restored the integrity of the damaged cartilage and recovered the expression levels of both aggrecan and COMP. Furthermore, NP-2007 effectively attenuated the MMT-induced upregulation of catabolic markers, including MMP-3, MMP-13, and ADAMTS-5, as well as serum levels of the systemic biomarker COMP. In this study, the molecular mechanisms elucidated in IL-1β-stimulated SW1353 cells were consistently mirrored by the phenotypic outcomes in the MMT rat model. Specifically, the in vitro suppression of matrix-degrading enzymes (MMP-3, MMP-13, and ADAMTS-5) and the preservation of aggrecan and COL2A1 directly translated to the attenuated serum levels of these enzymes and the restored proteoglycan/COMP integrity observed in vivo. This strong alignment between cellular mechanisms and systemic efficacy highlights the potential of NP-2007 as a disease-modifying osteoarthritis drug (DMOAD) candidate rather than a mere palliative analgesic. Given that the MMT model closely mimics the rapid catabolic cascade of human post-traumatic osteoarthritis (PTOA), NP-2007 could be highly effective in mitigating early-to-mid-stage joint degeneration following injury. However, because human chronic OA develops insidiously over decades and is heavily influenced by aging and metabolic factors, further long-term evaluations in aged spontaneous OA models and clinical trials remain essential to fully validate its therapeutic utility. Despite these promising findings, several limitations of the present study should be acknowledged. First, although we observed significant suppression of the Wnt/β-catenin and PI3K/Akt/mTOR signaling pathways, we did not employ specific pathway inhibitors to establish a direct causal link. Therefore, the observed chondroprotective effects are correlative, and further investigations are warranted to confirm the exact molecular dependency of NP-2007 on these pathways. Second, while the SW1353 cell line is a widely used model for OA research, its chondrosarcoma origin may not fully replicate the metabolic profile of primary human articular chondrocytes. Third, the sample size in our in vivo study was relatively small (*n* = 5 per group), and no formal a priori power calculation was performed. Although consistent with previous MMT-induced OA models, caution should be exercised when generalizing these histological and serum biomarker outcomes, and future studies with larger cohorts and pharmacokinetic analyses would further strengthen the translational relevance of NP-2007. Additionally, due to commercial confidentiality, the exact technical identities of the industrial enzymes and the precise composition of the mobile phase solvents used in LC-MS/MS analysis could not be disclosed. Although the definitive amino acid sequences of the ten major peptide fragments were successfully documented for structural reproducibility, the restriction on publishing the proprietary solvent formulation remains a limitation of this study. Lastly, a direct comparison with other established disease-modifying OA drug candidates was not conducted in this study. Addressing these limitations will be the focus of our future research to rigorously validate the clinical potential of NP-2007.

Collectively, these results demonstrate the chondroprotective efficacy of NP-2007, which appears to function by favorably modulating metabolic pathways within the osteoarthritic joint microenvironment. These findings provide promising preclinical evidence that NP-2007 holds the potential to attenuate structural joint degeneration, rather than merely offering temporary symptomatic relief. Nevertheless, rigorous clinical investigations remain indispensable to validate whether these preclinical structural benefits successfully translate into therapeutic outcomes in human patients.

## 4. Materials and Methods

### 4.1. Preparation of Enzyme-Treated Cervus elaphus L. Extract

*Cervus elaphus* L. was crushed (<10 mm) and subjected to water extraction with a 5-fold volume of purified water at 95–99 °C for 3 h, which was repeated for six cycles. The concentrated extract underwent a standardized, two-step enzymatic hydrolysis under strict industrial quality control protocols by Hanpoong Pharmaceutical Co., Ltd. (Jeonju, Korea): first with a food-grade endoprotease and subsequently with an exopeptidase. Each enzymatic reaction was performed at 50 ± 5 °C for 2 h, followed by heat inactivation at 90 °C for 0.5 h. To stabilize the hydrolysate, dextrin was added at a 9:1 (w/w) ratio. The mixture was vacuum-dried and pulverized to obtain the final enzyme-treated *cervus elaphus* L. extract (NP-2007) powder. To ensure batch-to-batch consistency, the peptide profile of NP-2007 was characterized using an LC-MS/MS setup consisting of a Vanquish UHPLC system and a Q Exactive Plus mass spectrometer (Thermo Fisher Scientific, Waltham, MA, USA) equipped with an AdvanceBio Peptide Mapping column (120 Å, 2.1 × 100 mm, 2.7 μm). Separation was performed under a proprietary mobile phase gradient optimized by Hanpoong Pharmaceutical Co., Ltd. De novo sequencing of the ten highest peaks confirmed the following collagen fragments: GR/RG, GPR, GDRGDAGPK, PGAGPR, GPVG, GMOGEGR, GPAGERGSOGPAGPK, GPRGPSGPQG, GPOGPAGPR, and GPAGPAGPR (where O denotes hydroxyproline). Among these, GPAGERGSOGPAGPK was the predominant constituent based on peak area integration, serving as the primary chemical marker for quality control.

### 4.2. Cell Culture and Stabilization

The human chondrosarcoma cell line SW1353 was obtained from the American Type Culture Collection (ATCC; Manassas, VA, USA). To ensure phenotypic stability and verify growth characteristics, the cells were initially stabilized in Leibovitz’s L-15 medium (ATCC) supplemented with 10% fetal bovine serum (FBS; Hyclone, Logan, UT, USA) and 1% penicillin/streptomycin (P/S; 100 U/mL penicillin and 100 mg/mL streptomycin) at 37 °C in a non-CO_2_ incubator, following the manufacturer’s instructions. For the experimental phase, the culture medium was transitioned to Dulbecco’s modified Eagle’s medium (Hyclone) containing 10% FBS and 1% P/S at 37 °C and 5% CO_2_ incubator. This transition was conducted to align with established protocols for IL-1β-induced inflammatory models in SW1353 cells and to evaluate chondroprotective effects under standardized conditions [39]. The cells were further cultivated in this medium prior to their use in subsequent experiments.

### 4.3. Cell Viability Assay

SW1353 cells were seeded in 96-well plates at a density of 5 × 10^3^ cells/well and incubated in complete medium for 24 h to allow cell attachment. Subsequently, the culture medium was removed and replaced with 100 μL of serum-free medium supplemented with various concentrations of NP-2007 (100, 250, and 500 μg/mL). After an additional 24 h of incubation, cell viability was evaluated using the EZ-CYTOX kit (DoGenBio, Seoul, Korea) according to the manufacturer’s instructions. Absorbance was measured at 450 nm using a microplate reader (Thermo Scientific, Waltham, MA, USA). These concentrations were selected based on preliminary screening and previous literature on collagen hydrolysates to evaluate dose-dependent efficacy within a non-cytotoxic range [13].

### 4.4. ELISA Analysis

For the in vitro study, SW1353 cells were pre-stimulated with IL-1β (10 ng/mL) for 2 h, followed by the addition of NP-2007 (100, 250, and 500 μg/mL) directly to the medium without media exchange to maintain sustained inflammatory conditions. Culture supernatants were collected after 24 h of treatment to measure IL-6, TNF-α, ADAMTS5, MMP-3, and MMP-13, while the supernatant collected after 72 h of treatment was used for aggrecan analysis. All analytes were measured using human ELISA kits (R&D Systems, Minneapolis, MN, USA) according to the manufacturer’s instructions. Absorbance was measured at 450 nm using a microplate reader (Thermo Scientific, Waltham, MA, USA), and each sample was measured in triplicate.

For the in vivo study, serum collected from rats at the end of the experiment was used for ELISA analysis. Serum MMP-3 and MMP-13 concentrations were measured using Abcam (Cambridge, UK) and Elabscience (Houston, TX, USA) kits, respectively. In addition, ADAMTS5 and COMP were measured using Antibodies.com (Cambridge, UK) and MyBioSource (San Diego, CA, USA) kits, respectively. All analyses were performed according to the manufacturers’ instructions, and absorbance was measured at 450 nm using a microplate reader (Thermo Scientific, Waltham, MA, USA). Serum samples from five animals per group were analyzed individually.

### 4.5. Western Blotting Analysis

Total protein was extracted from the cells using a lysis buffer supplemented with a protease inhibitor cocktail (Thermo Scientific, Rockford, IL, USA). Protein concentrations were determined via the Pierce™ BCA Protein Assay Kit (Thermo Scientific) according to the manufacturer’s protocol. Standardized protein samples (30 μg per lane) were resolved by 10% sodium dodecyl sulfate–polyacrylamide gel electrophoresis and subsequently electrotransferred to polyvinylidene difluoride membranes using the Trans-Blot Turbo Transfer System (Bio-Rad, Hercules, CA, USA). The membranes were blocked with 5% skim milk in Tris-buffered saline with Tween 20 (TBST) for 1 h at room temperature to prevent non-specific binding and then incubated overnight at 4 °C with primary antibodies (1:1000) targeting the following markers: iNOS (cat#20609), COX2 (cat#ab15191), COL2A1 (cat#43306), β-catenin (cat#8480), p-GSK3-β (cat#9336), GSK3-β (cat#9315), TCF4/TCF7L2 (cat#2565), LEF1 (cat#2286), SOX9 (cat#82630), p-PI3K (cat#4228), PI3K (cat#4257), p-AKT (cat#9271), AKT (cat#9272), p-mTOR (cat#2971), and mTOR (cat#2983). β-actin (1:10,000; cat#3700) was employed as the internal loading control. Following three washes with TBST, the membranes were probed with horseradish peroxidase (HRP)-conjugated secondary antibodies for 2 h at room temperature. The immunoreactive bands were developed using an Amersham ECL kit and visualized with an Amersham Imager 600 (GE Healthcare, Buckinghamshire, UK). For densitometric quantification via ImageJ software (version 1.54g, National Institutes of Health, Bethesda, MD, USA), target bands were accurately selected and distinguished from non-specific background signals or multiple bands (particularly for LEF1, SOX9, and p-PI3K) by strictly cross-referencing the expected molecular weights specified in the manufacturers’ antibody datasheets with the migrated positions of the protein molecular weight size markers.

For each target protein, samples were analyzed using separate SDS-PAGE gels run in parallel and transferred onto individual PVDF membranes; membranes were not stripped and reprobed. A dedicated β-actin loading control was included for each corresponding gel and membrane setup. Western blot experiments were independently repeated three times using biological replicates, and band intensities were quantified using ImageJ software. Total protein expression levels were normalized to the corresponding β-actin signal, whereas phosphorylated forms were normalized to their respective total proteins to determine the expression ratios. Representative blot images shown in the figures were selected from experiments yielding consistent results. Full uncropped Western blot images, including molecular weight markers and lane boundaries, are provided in the Supplementary Materials. No lanes were removed, rearranged, or spliced during figure assembly.

### 4.6. Animal Study

This study was designed and reported in strict accordance with the ARRIVE Essential 10 guidelines to ensure experimental transparency and reproducibility. All experimental procedures were approved by the Institutional Animal Care and Use Committee (IACUC) of the Jeonju AgroBio-Materials Institute (Approval No. JAMI IACUC 2025005).

Seven-week-old male Sprague–Dawley (SD) rats (*n* = 20) were subjected to MMT surgery to induce OA at Japan SLC, Inc. (Shizuoka, Japan). Following a one-week post-operative recovery period at the surgical facility, the MMT-induced rats (*n* = 20) and age-matched normal SD rats (*n* = 5) from Japan SLC were imported and purchased through the Central Laboratory Animal Center (Central Lab Animal Inc., Seoul, Korea). Upon arrival, all animals were acclimated to a climate-controlled environment (22 ± 2 °C, 55 ± 5% humidity, 12 h light/dark cycle) for one week with *ad libitum* access to food and water prior to the experiments. In this study, an individual rat was defined as the experimental unit.

MMT-induced rats (*n* = 20) were randomly assigned to one of four experimental groups (*n* = 5 per group). Age-matched normal rats (*n* = 5) were non-randomly assigned to the normal group as they did not undergo the surgical procedure. Normal SD rats were assigned to the normal group and received distilled water as a vehicle. MMT-induced OA rats were further partitioned into four groups: a control group administered distilled water, a positive control group treated with 300 mg/kg/day of MSM, and two experimental groups receiving NP-2007 at doses of 50 and 200 mg/kg/day, respectively. These in vivo doses were selected based on our previous findings demonstrating the anti-inflammatory and anti-osteoarthritic efficacy of NP-2007 within this range in a different rodent model [13]. MSM was employed as a positive control due to its established efficacy in alleviating articular inflammation and preserving cartilage integrity in experimental OA models [40]. The sample size (*n* = 5 per group) was determined based on the 3Rs principle for animal ethics (Reduction) and is consistent with established precedents in the MMT-induced OA model, which have demonstrated that this number is sufficient to achieve statistical significance in primary histological and biochemical outcomes [17]. All treatments were administered via oral gavage once daily for the duration of the experimental period following the one-week post-surgical recovery phase. Animals were monitored once per week for body weight and signs of distress. This stratified dosing regimen was implemented to evaluate the potential dose-dependent chondroprotective efficacy of NP-2007 against surgically induced OA progression.

Animals within the same experimental group were housed in two separate cages to prevent cross-group social interaction and ensure stable husbandry conditions. All 25 animals were accounted for in the final results, and no data points were excluded during the study period. Investigators were not blinded during treatment administration; however, to mitigate observer bias, outcome assessments—including histological scoring of joint tissues and systemic blood analysis—were performed post-mortem using coded samples and objective measurement criteria. The primary outcome measure was the histopathological analysis of knee joint tissue, while the secondary outcome measures were body weight, IHC analysis of knee joint tissue, and serum levels of matrix-degrading enzymes and structural biomarkers.

### 4.7. Histological Analysis

For histological assessment, harvested knee joint tissues were fixed in 4% paraformaldehyde and decalcified prior to paraffin embedding. The decalcified tissues were trimmed to approximately 2–3 mm in thickness, placed in labeled tissue cassettes, and subjected to routine tissue processing for 13 h. The processed tissues were then sectioned at a thickness of approximately 3 μm, mounted onto glass slides, dried, deparaffinized, rehydrated, and rinsed with distilled water. To evaluate the overall morphological alterations, sections were stained with H&E. Additionally, Safranin O staining was performed to assess the distribution and content of proteoglycans within the articular cartilage. The stained specimens were visualized and captured using an optical microscopic system (Olympus, Tokyo, Japan).

### 4.8. IHC Analysis

For IHC staining, paraffin-embedded tissue sections were cut at a thickness of 4 μm, deparaffinized, rehydrated, and rinsed with distilled water. Endogenous peroxidase activity was blocked using Peroxide Blocking Reagent (DAKO, Agilent Technologies, Santa Clara, CA, USA) for 10 min at room temperature. After washing with phosphate-buffered saline, the sections were incubated overnight at 4 °C with primary antibodies against COMP (PA5-95547; Thermo Fisher Scientific, Waltham, MA, USA) and aggrecan (BS-11655R; Thermo Fisher Scientific, Waltham, MA, USA). After additional washing with DAKO wash buffer, the sections were incubated with EnVision+ System-HRP (DAKO, Agilent Technologies, USA) for 30 min at room temperature and developed with 3,3-diaminebenzidine tetrachloride for 3 min. Finally, slides were counterstained with Mayer’s hematoxylin, dehydrated, cleared, and mounted.

### 4.9. Statistical Analysis

All experimental data are expressed as the mean ± standard deviation. Statistical analyses were performed using GraphPad Prism software (version 5; GraphPad Software, San Diego, CA, USA). Significant differences between groups were determined using one-way analysis of variance, followed by Tukey’s post hoc test for multiple comparisons. Prior to the ANOVA, the normality of the data distribution was rigorously assessed using the Kolmogorov–Smirnov test, and the homogeneity of variance was verified using Levene’s test. All experimental groups exhibited a *p*-value > 0.05, confirming that the data satisfied the normality assumptions required for parametric analysis. To account for the potential inflation of the Type I error rate arising from testing multiple related endpoints across various cytokines, matrix-degrading enzymes, serum biomarkers, and Western blot targets, Tukey’s post hoc test was strictly applied. This multiple-comparison approach effectively controls the family-wise error rate, thereby ensuring the statistical validity and adequacy of the comparisons. A *p*-value of less than 0.05 was considered the threshold for statistical significance.

## 5. Conclusions

This study demonstrates that NP-2007, a specialized enzyme-treated low-molecular-weight collagen derived from *Cervus elaphus* L., modulates selected catabolic and anabolic markers within the osteoarthritic joint microenvironment. Our in vitro findings in SW1353 cells reveal that NP-2007 effectively mitigates the IL-1β-induced inflammatory response and ECM degradation. These protective actions are associated with changes in Wnt/β-catenin and PI3K/Akt/mTOR signaling pathways, subsequently restoring the expression of the master chondrogenic factor SOX9. Furthermore, the therapeutic efficacy of NP-2007 was validated in a surgically induced MMT rat model, which closely mimics the structural and pathophysiological progression of human PTOA. Oral administration of NP-2007 considerably preserved articular cartilage integrity, maintained proteoglycan content, and reduced systemic catabolic biomarkers, including serum COMP levels. These findings provide robust evidence for the potential of NP-2007 as a functional intervention for the long-term management and structural preservation of osteoarthritic joints.

## Data Availability

The raw data supporting the conclusions of this article will be made available by the authors on request.

## Supplementary Materials

The following supporting information can be downloaded at: https://www.mdpi.com/article/10.3390/ijms27135785/s1.

## Abbreviations

The following abbreviations are used in this manuscript:

ADAMTS-5	A disintegrin and metalloproteinase with thrombospondin motifs-5
ATCC	American Type Culture Collection
COL2A1	Collagen type II alpha 1 chain
COMP	Cartilage oligomeric matrix protein
COX-2	Cyclooxygenase-2
ECM	Extracellular matrix
ELISA	Enzyme-linked immunosorbent assay
FBS	Fetal bovine serum
H&E	Hematoxylin and eosin
HRP	Horseradish peroxidase
IACUC	Institutional Animal Care and Use Committee
IHC	Immunohistochemical
IL-1β	Interleukin-1β
IL-6	Interleukin-6
iNOS	Inducible nitric oxide synthase
MIA	Monosodium iodoacetate
MMT	Medial meniscal transection
MMP	Matrix metalloproteinase
MSM	Methylsulfonylmethane
OA	Osteoarthritis
PTOA	Post-traumatic osteoarthritis
SD	Sprague–Dawley
TNF-α	Tumor necrosis factor-α
