# Supplementary material for: Chondroprotective Effects of Enzyme-Treated Extract from Cervus elaphus L. in a Rat Model of Osteoarthritis
Source: Int J Mol Sci. 2026 Jun 26;27(13):5785. doi: 10.3390/ijms27135785 (PMC13361236; doi:10.3390/ijms27135785)

These are the molecular weight size markers used in this study.

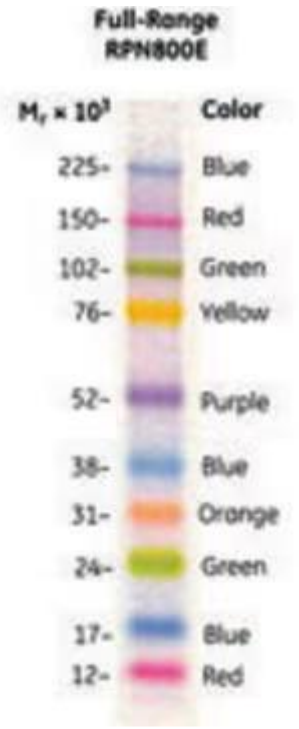

Cytiva RPN800E

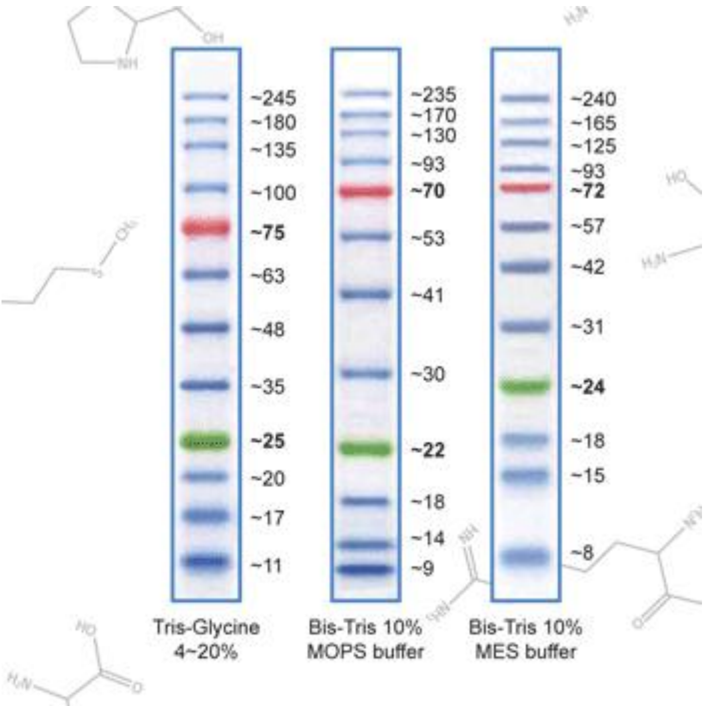

ZEPTO, BB0312

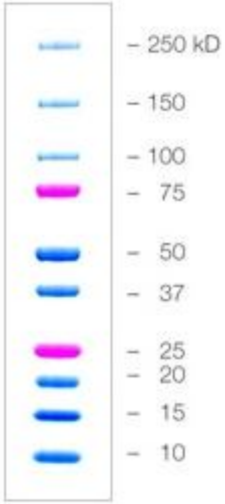

BIO-RAD, 1610374

Figure 1. (b) iNOS (130 kDa)

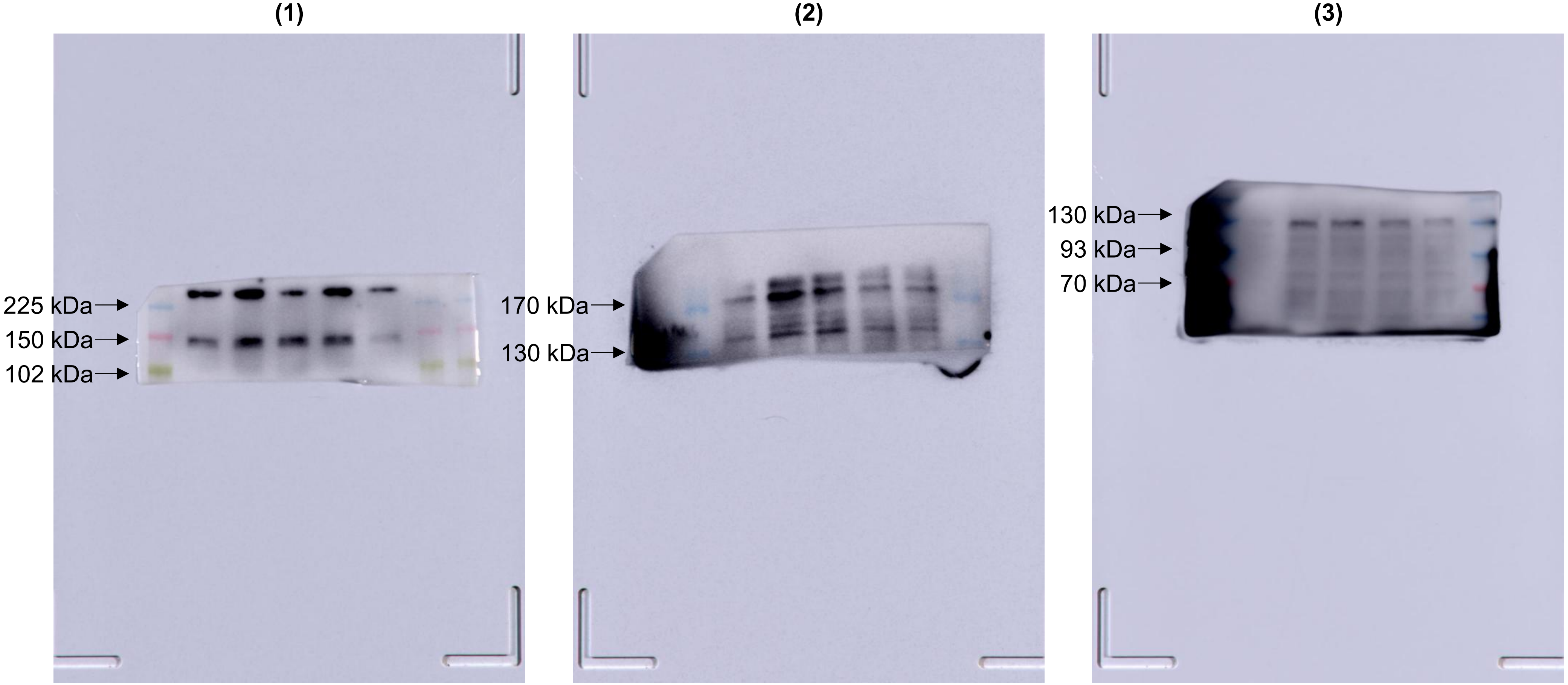

Figure 1. (b) COX-2 (74 kDa)

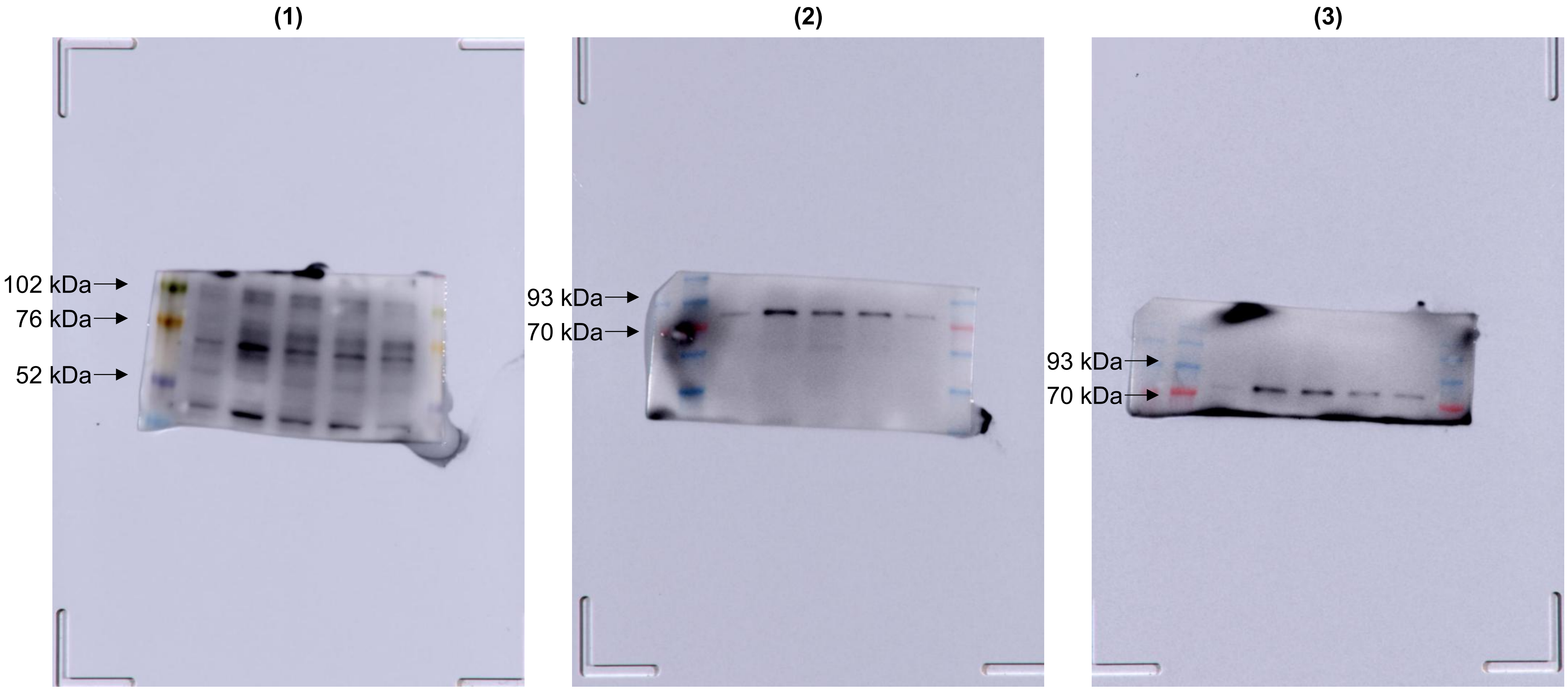

Figure 1. (b) β-actin (45 kDa)

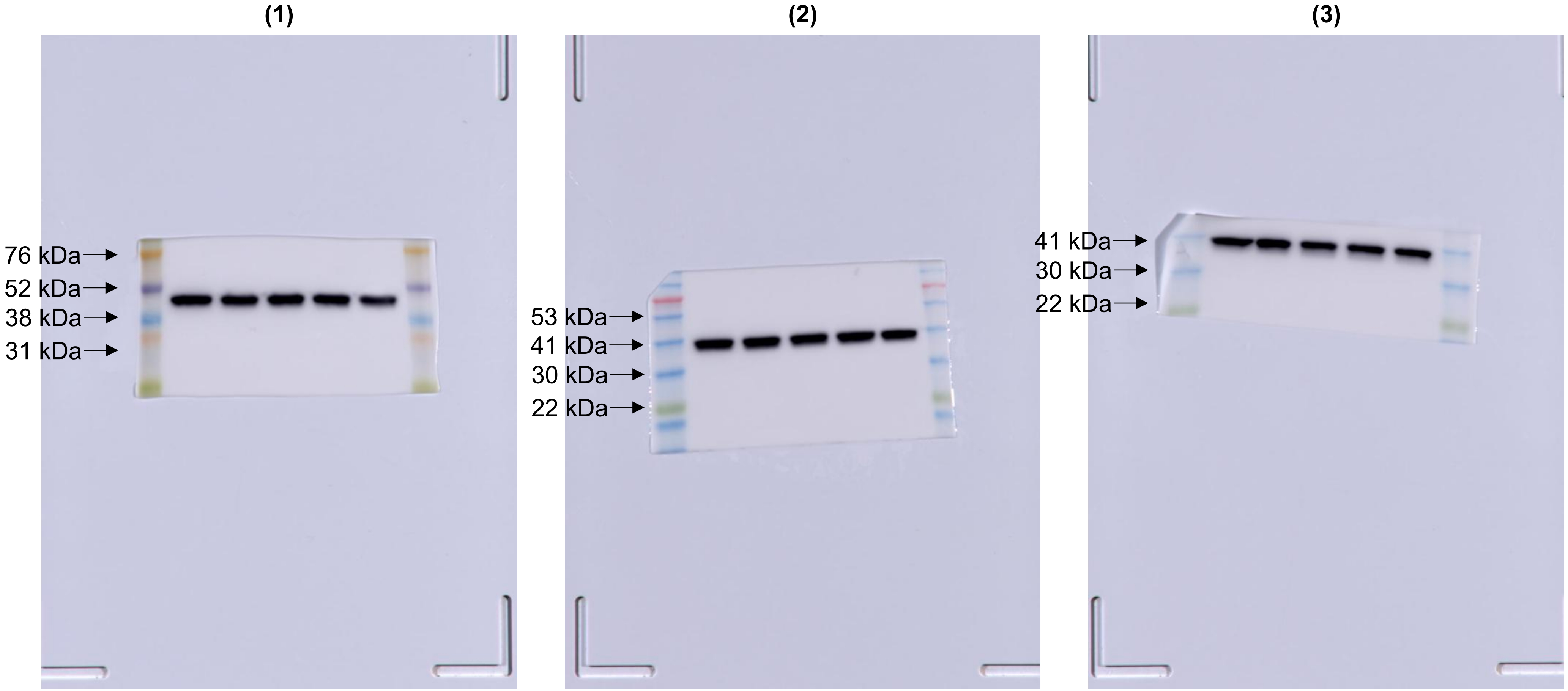

Figure 2. (e) COL2A1 (200 kDa)

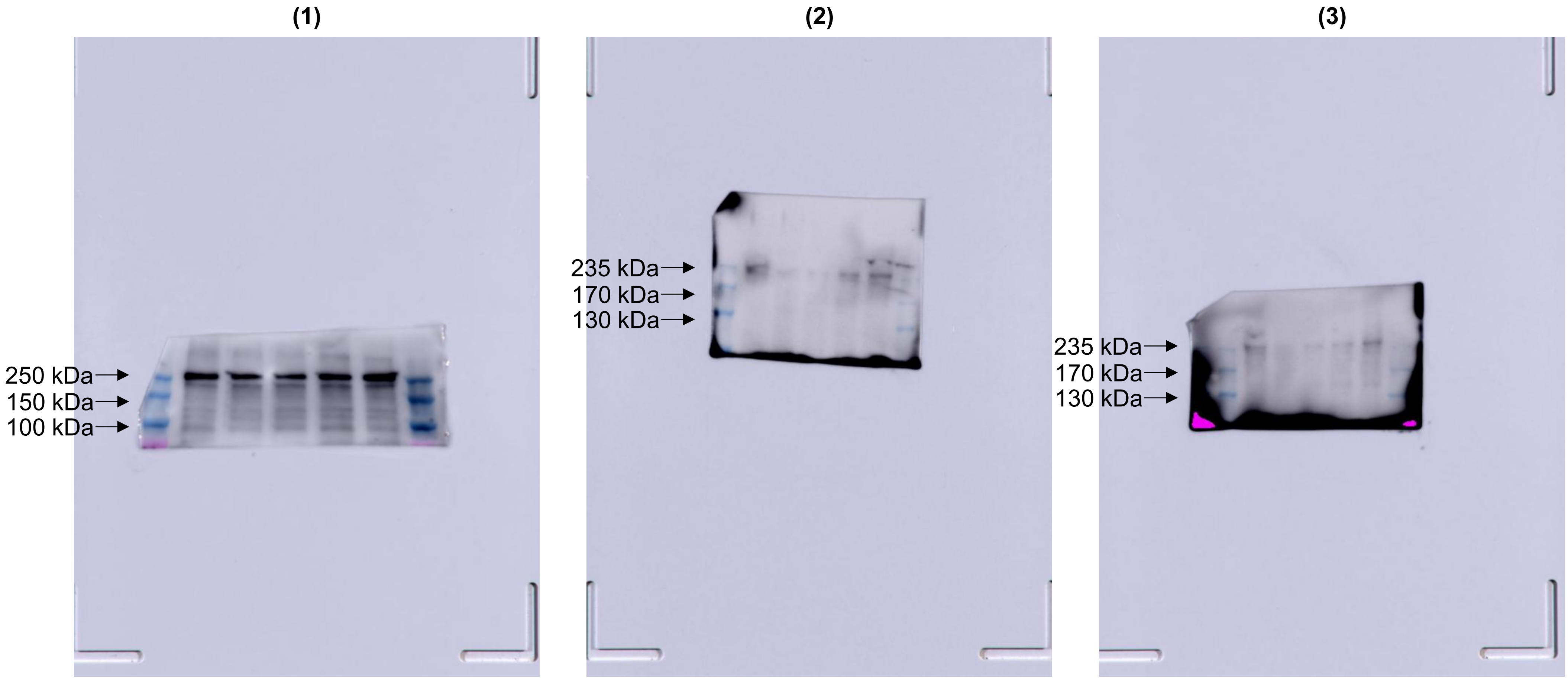

Figure 2. (e) β-actin (45 kDa)

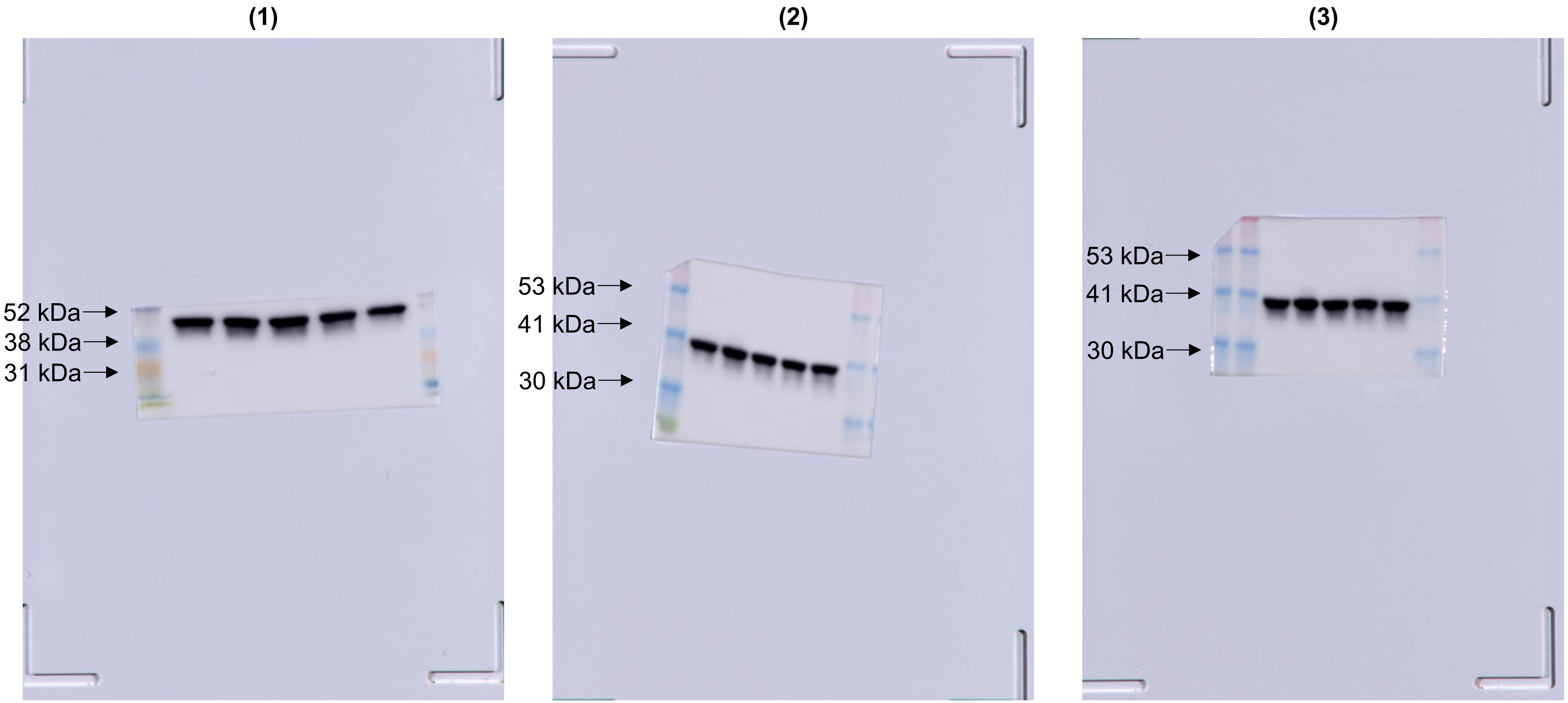

Figure 3. (a) β-catenin (92 kDa)

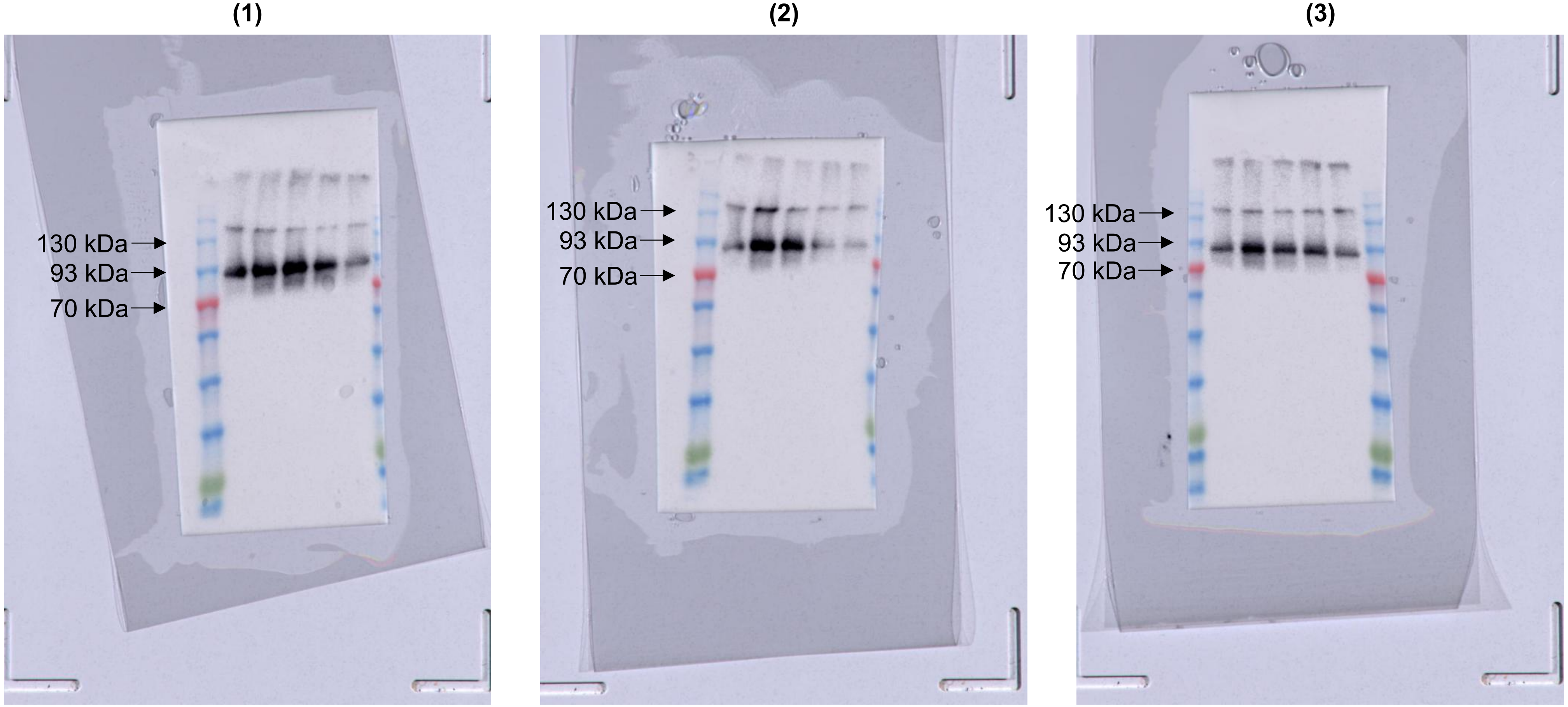

Figure 3. (a) p-GSK3β (46kDa)

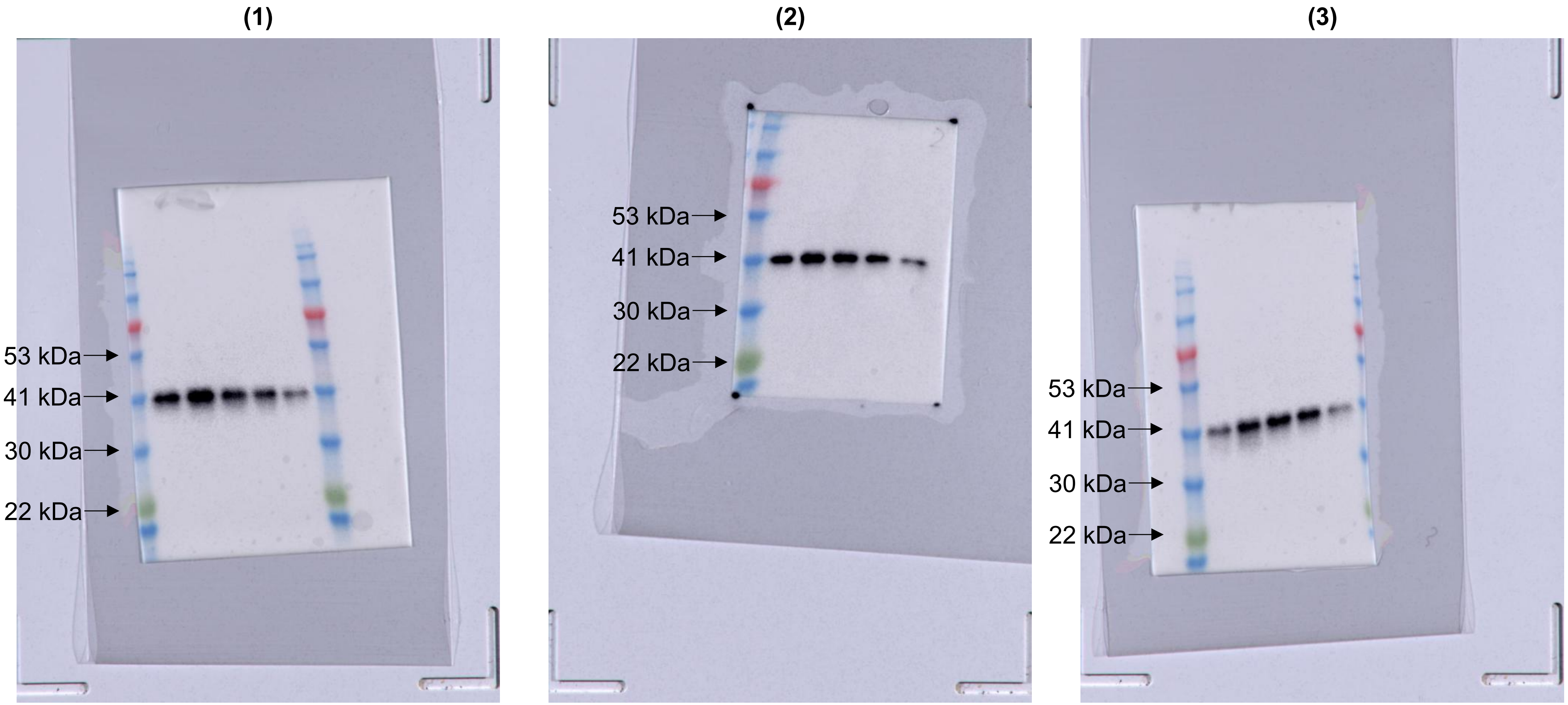

Figure 3. (a) GSK3β (46 kDa)

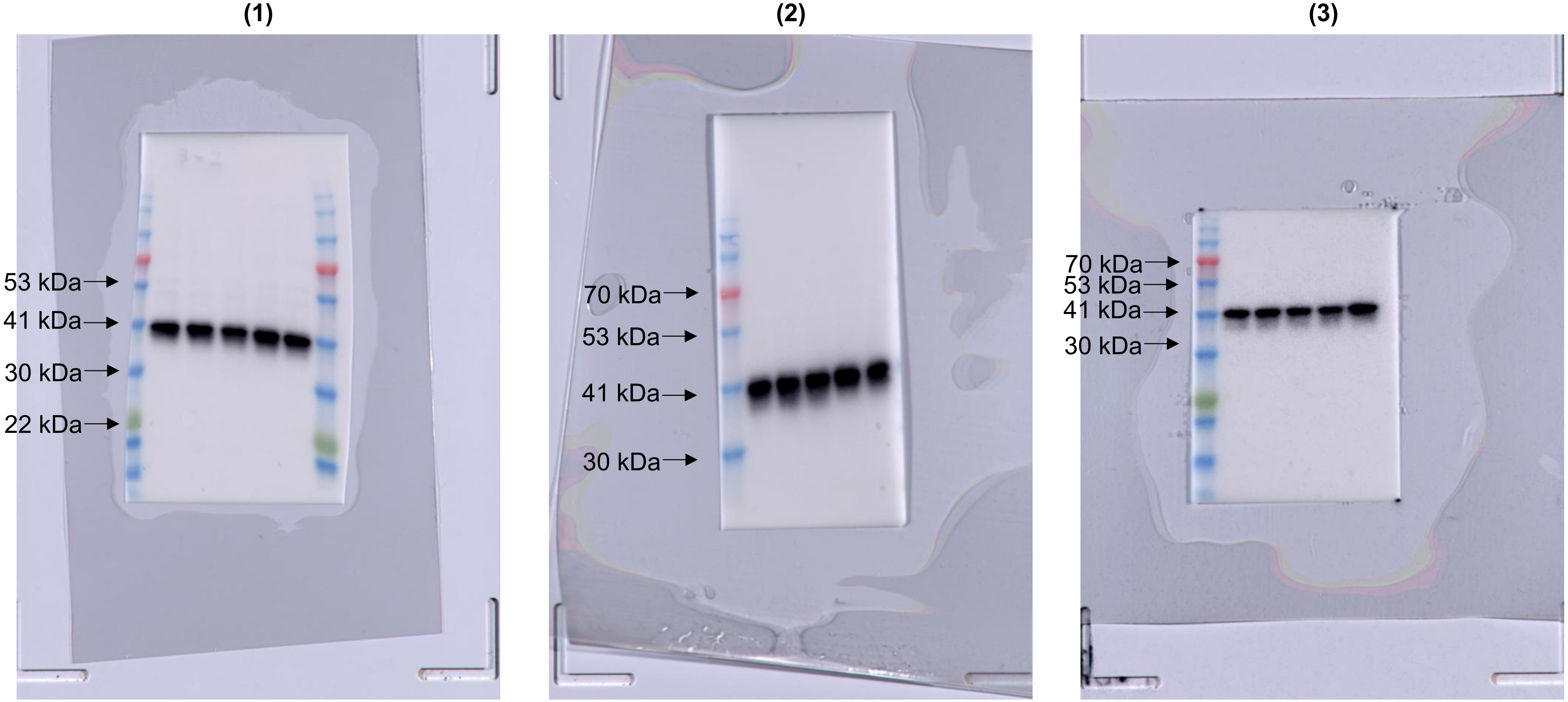

Figure 3. (a) TCF4/TCF7 (58, 79 kDa)

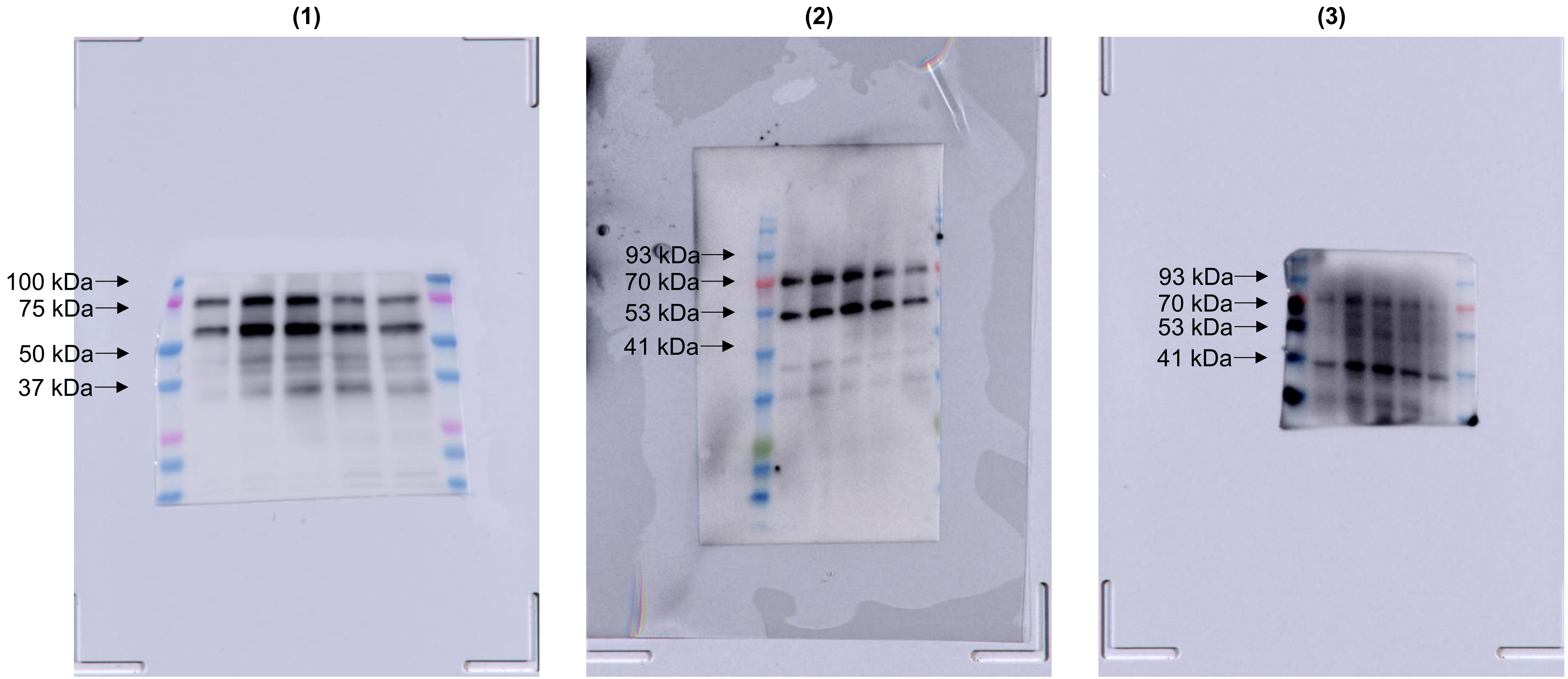

Figure 3. (a) LEF1 (25-58 kDa)

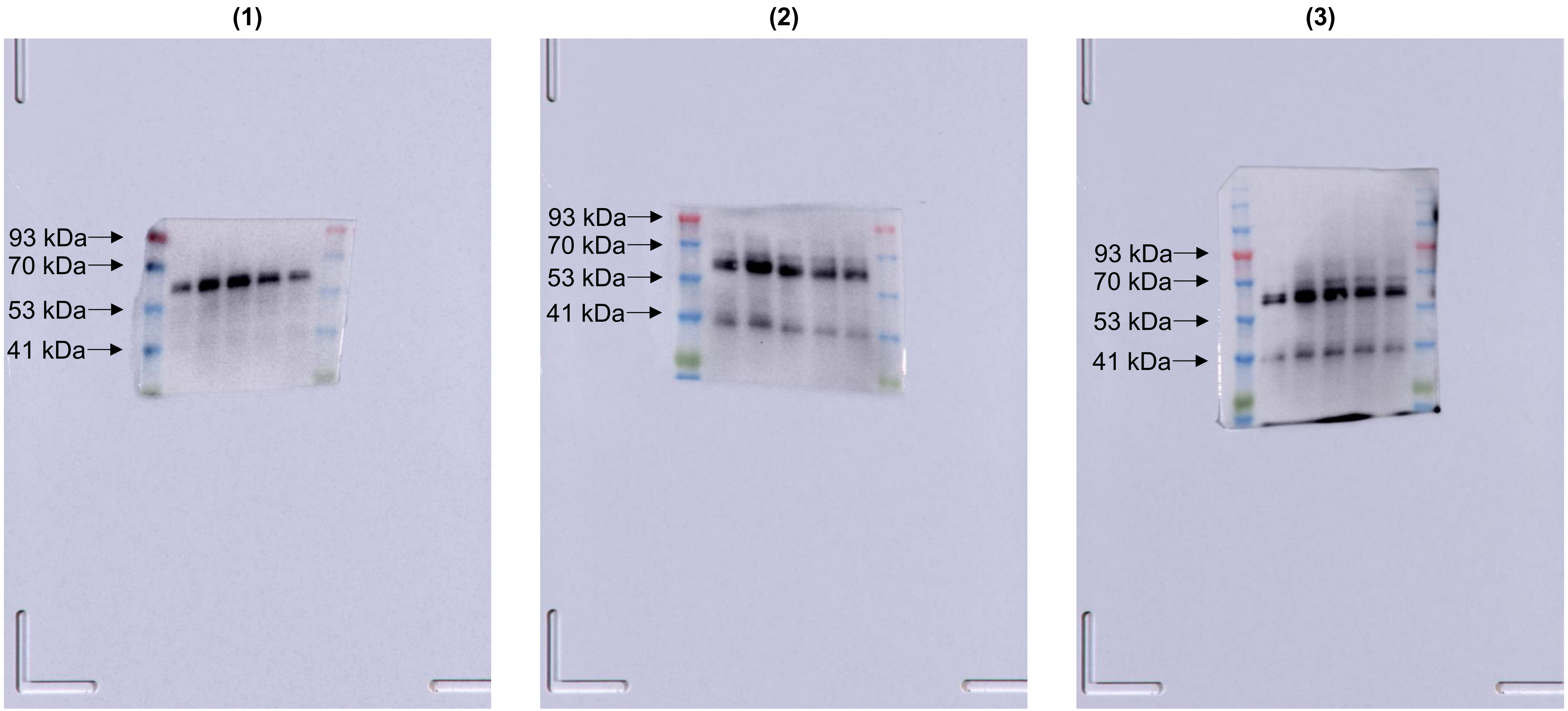

Figure 3. (a) SOX9 (70 kDa)

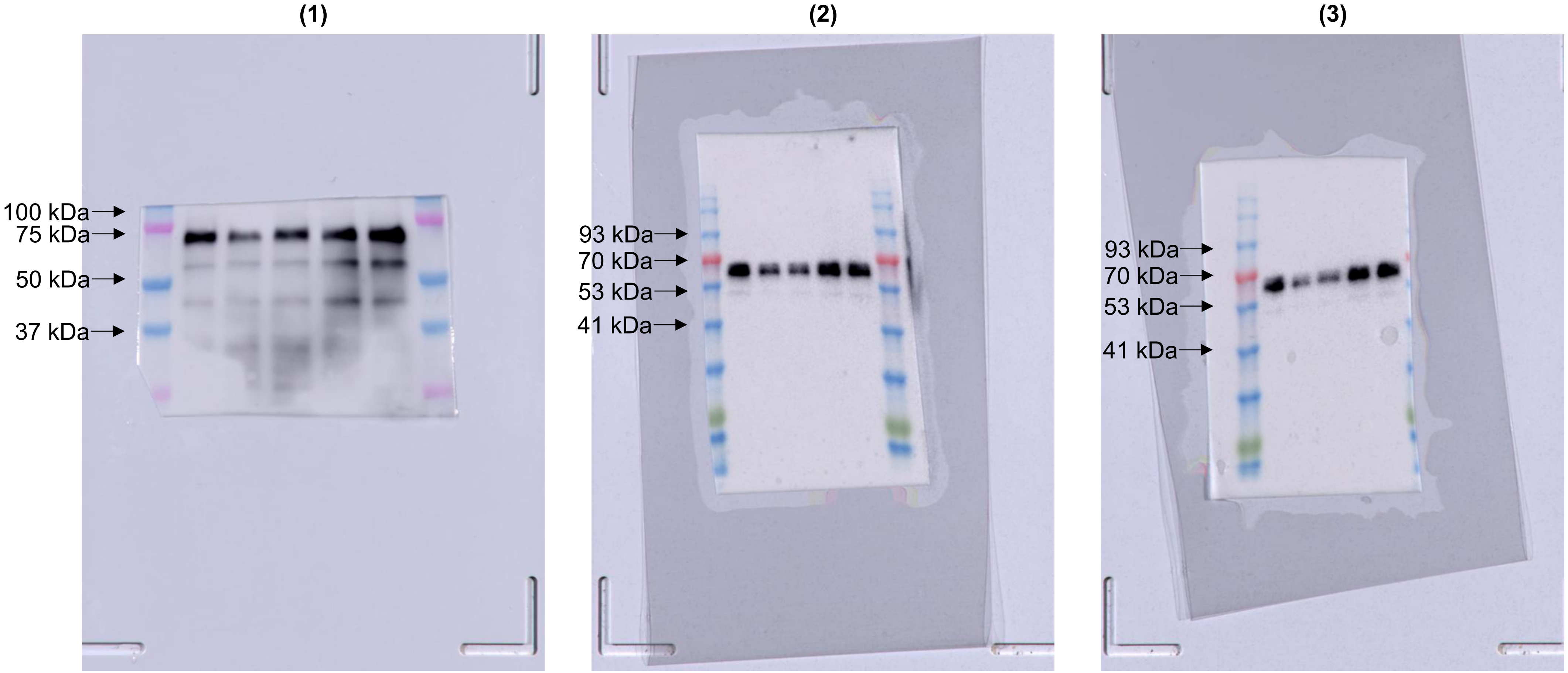

Figure 3. (a) β-actin (45 kDa)

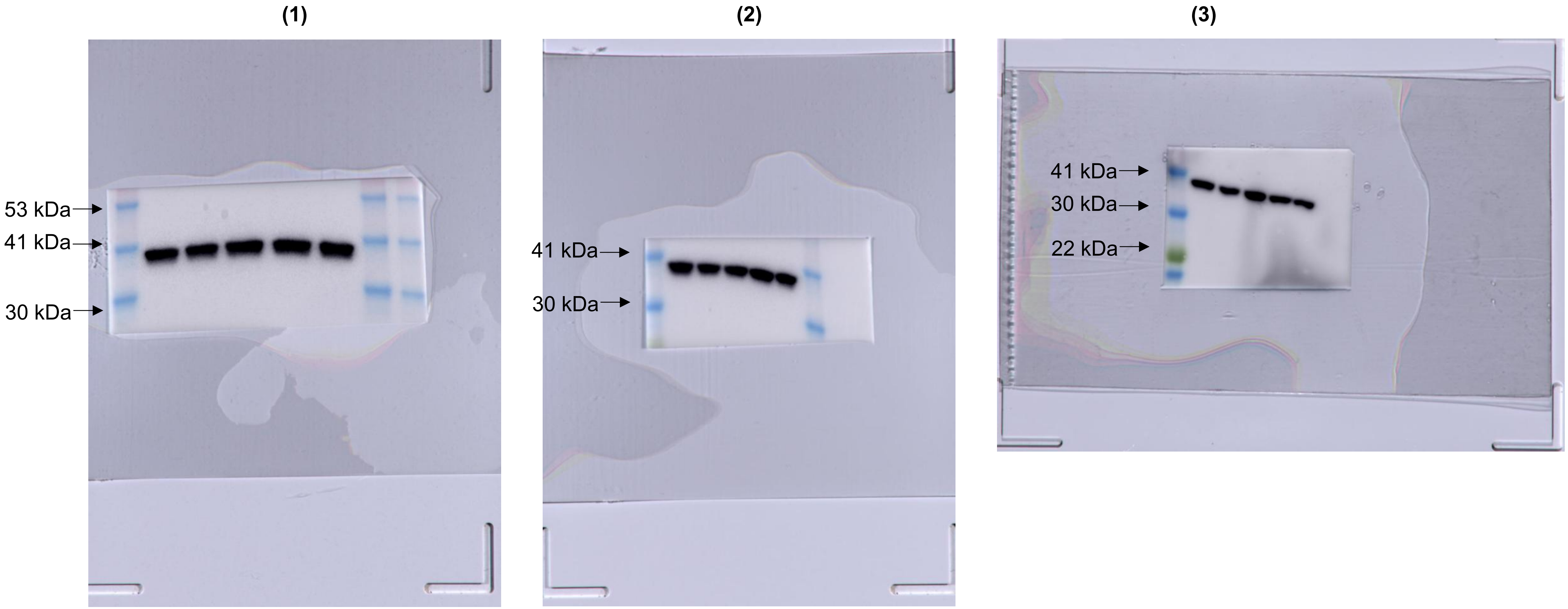

Figure 3. (b) p-PI3K (85 kDa)

(1)

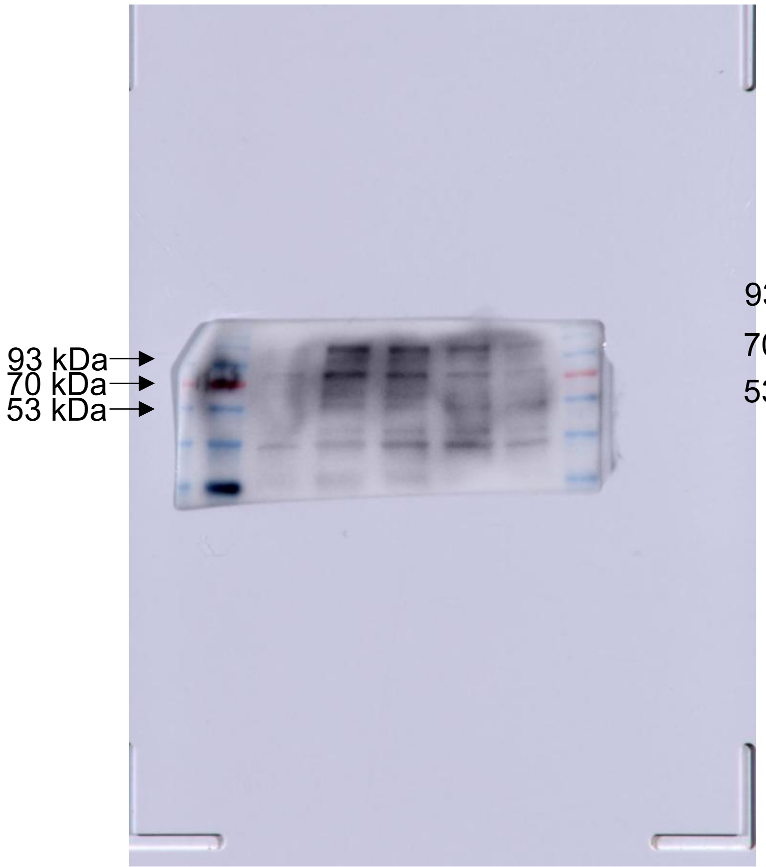

(2)

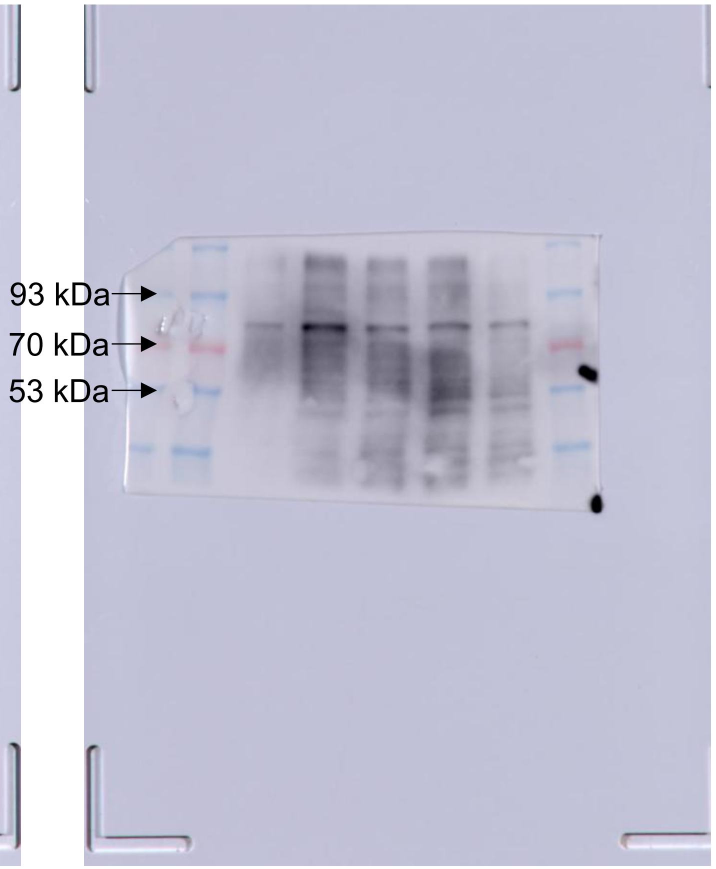

(3)

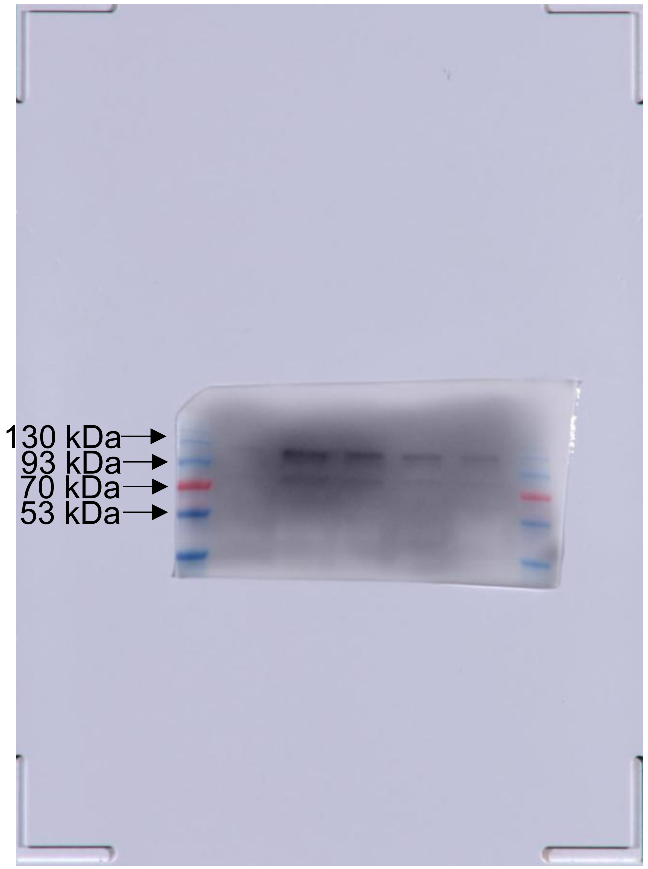

Figure 3. (b) PI3K (85 kDa)

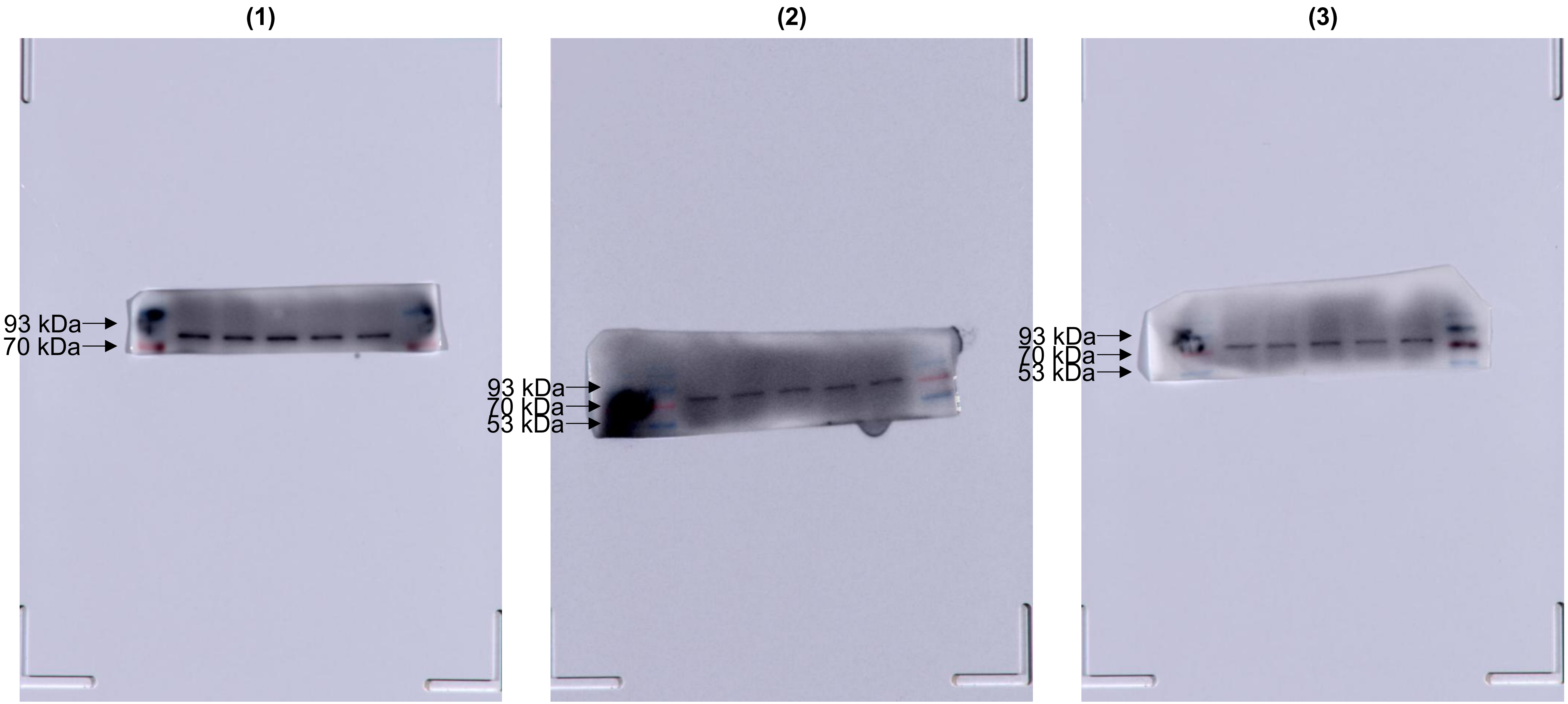

Figure 3. (b) p-AKT (60 kDa)

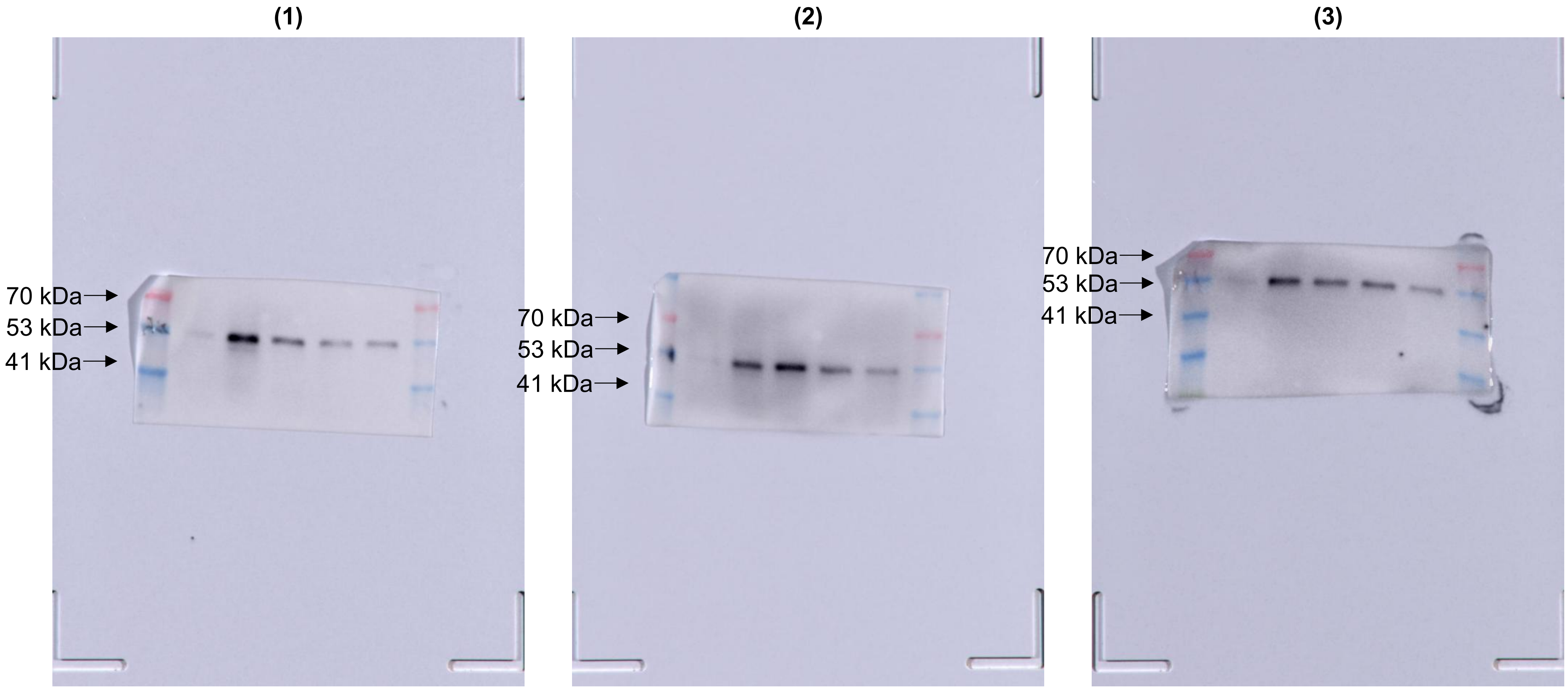

Figure 3. (b) AKT (60 kDa)

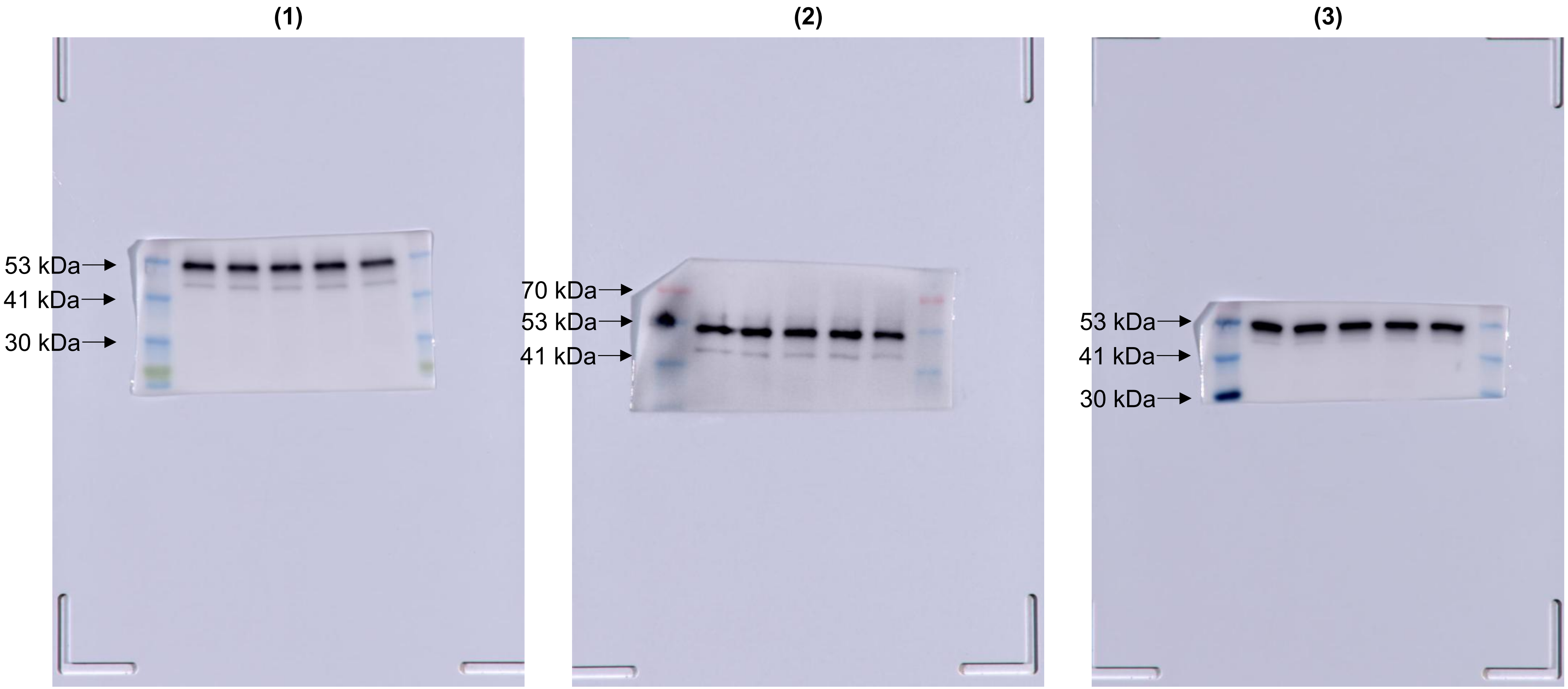

Figure 3. (b) p-mTOR (289 kDa)

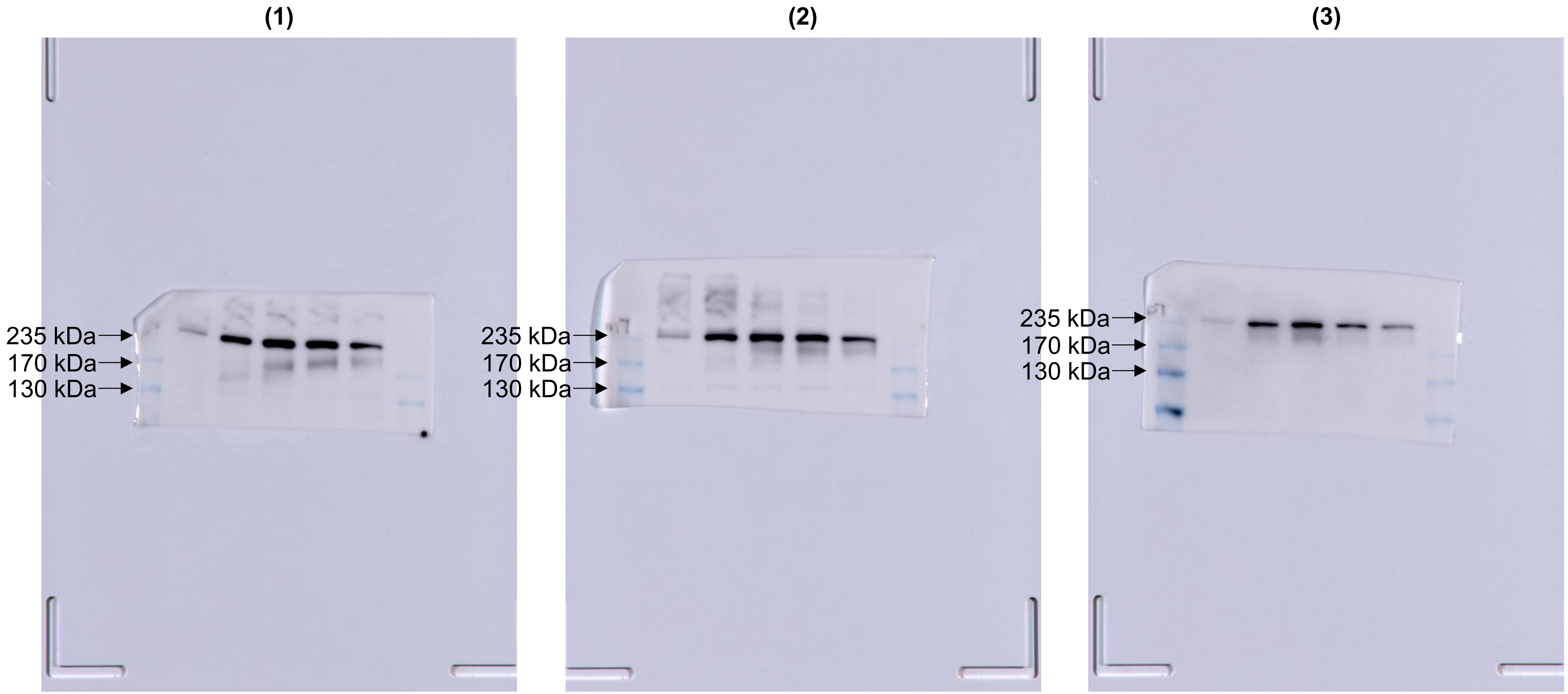

Figure 3. (b) mTOR (289 kDa)

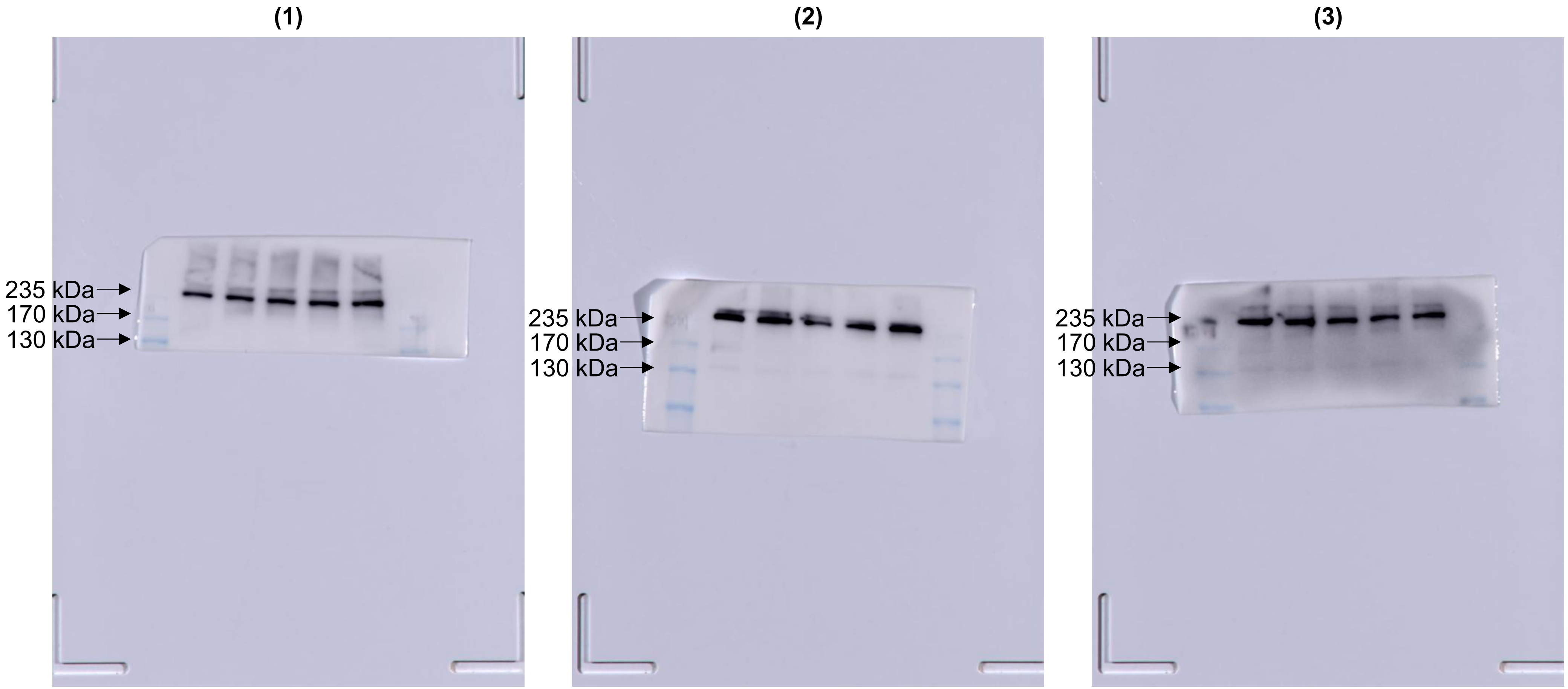

Figure 3. (b) β-actin (45 kDa)

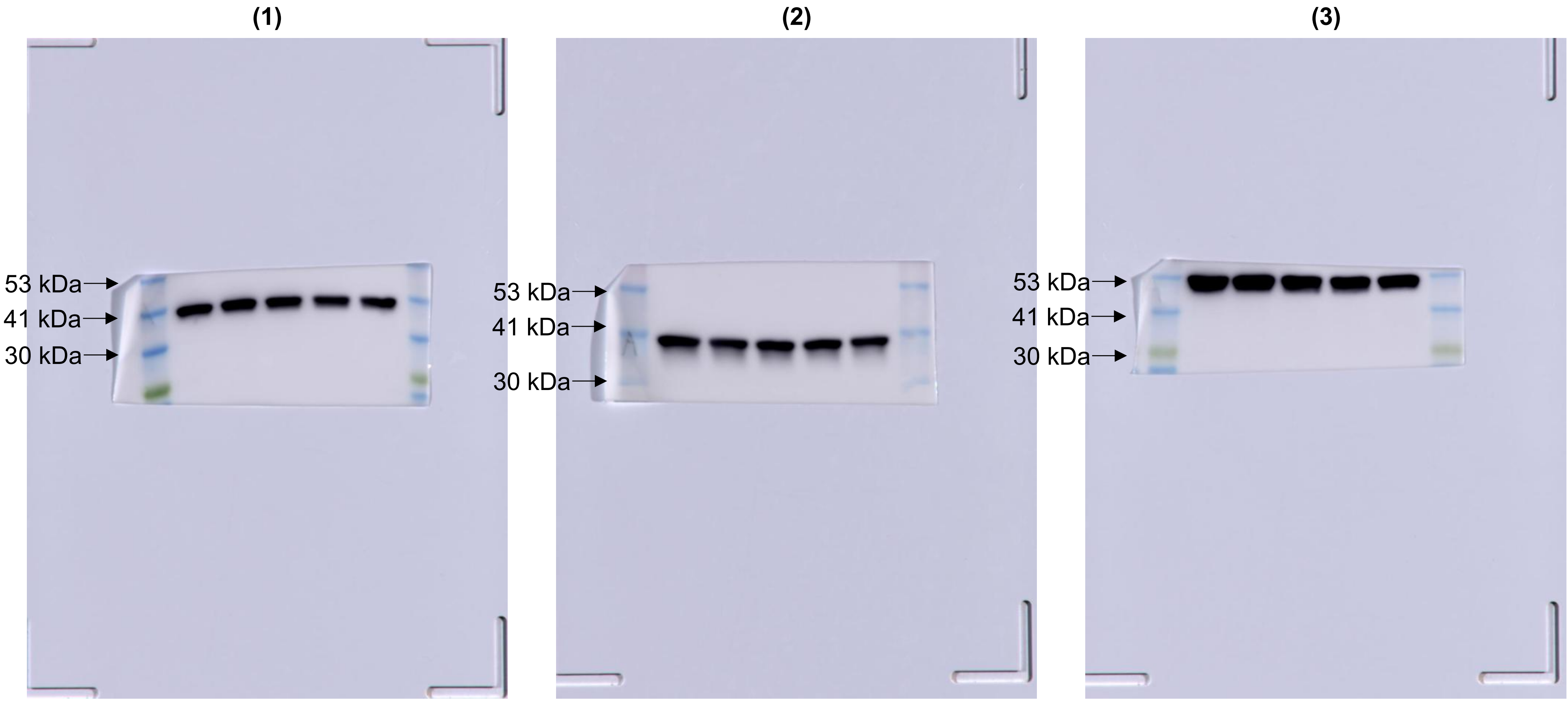

Supplement: Supplementary file 1 [file ijms-27-05785-s001.zip › ijms-4295174-supplementary.pdf]
